# Function of CSF1 and IL34 in Macrophage Homeostasis, Inflammation, and Cancer

**DOI:** 10.3389/fimmu.2019.02019

**Published:** 2019-09-04

**Authors:** WeiYu Lin, Daqi Xu, Cary D. Austin, Patrick Caplazi, Kate Senger, Yonglian Sun, Surinder Jeet, Judy Young, Donnie Delarosa, Eric Suto, Zhiyu Huang, Juan Zhang, Donghong Yan, Cesar Corzo, Kai Barck, Sharmila Rajan, Carrie Looney, Vineela Gandham, Justin Lesch, Wei-Ching Liang, Elaine Mai, Hai Ngu, Navneet Ratti, Yongmei Chen, Dinah Misner, Tori Lin, Dimitry Danilenko, Paula Katavolos, Estelle Doudemont, Hirdesh Uppal, Jeffrey Eastham, Judy Mak, Patricia E. de Almeida, Katherine Bao, Azadeh Hadadianpour, Mary Keir, Richard A. D. Carano, Lauri Diehl, Min Xu, Yan Wu, Robby M. Weimer, Jason DeVoss, Wyne P. Lee, Mercedesz Balazs, Kevin Walsh, Kathila R. Alatsis, Flavius Martin, Ali A. Zarrin

**Affiliations:** Genentech, South San Francisco, CA, United States

**Keywords:** CSF1 and Il34 inhibition, cancer, inflammation, macrophage, monocyte, CSF1R, microglia, Langerhans cells

## Abstract

Colony-stimulating factor 1 (CSF1) and interleukin 34 (IL34) signal *via* the CSF1 receptor to regulate macrophage differentiation. Studies in IL34- or CSF1-deficient mice have revealed that IL34 function is limited to the central nervous system and skin during development. However, the roles of IL34 and CSF1 at homeostasis or in the context of inflammatory diseases or cancer in wild-type mice have not been clarified *in vivo*. By neutralizing CSF1 and/or IL34 in adult mice, we identified that they play important roles in macrophage differentiation, specifically in steady-state microglia, Langerhans cells, and kidney macrophages. In several inflammatory models, neutralization of both CSF1 and IL34 contributed to maximal disease protection. However, in a myeloid cell-rich tumor model, CSF1 but not IL34 was required for tumor-associated macrophage accumulation and immune homeostasis. Analysis of human inflammatory conditions reveals IL34 upregulation that may account for the protection requirement of IL34 blockade. Furthermore, evaluation of IL34 and CSF1 blockade treatment during *Listeria* infection reveals no substantial safety concerns. Thus, IL34 and CSF1 play non-redundant roles in macrophage differentiation, and therapeutic intervention targeting IL34 and/or CSF1 may provide an effective treatment in macrophage-driven immune-pathologies.

## Introduction

Macrophages are multifunctional cell types that play critical roles in host defense, clearance of apoptotic cells, as well as tissue development, homeostasis, and repair (1). Fate mapping and genomic and functional studies suggest that macrophage differentiation is complex and influenced by environmental cues (2). Macrophage ontogeny includes resident populations that develop during embryogenesis independently of hemopoietic stem cells (HSCs) (3) as well as those that originate from bone marrow HSCs (4). Tissue-resident macrophages include splenic red-pulp macrophages, lung alveolar macrophages, epidermal Langerhans cells (LCs), brain microglia, liver Kupffer cells (KCs), large peritoneal macrophages, and F4/80-high pancreatic, kidney, and cardiac macrophages (5). Many tissue-resident macrophages are long-lived in mice and can proliferate within their tissue of residence, a mechanism involved in their maintenance (6–10). Nevertheless, bone marrow–derived progenitor cells also contribute to subsets that reside in the lamina propria, spleen, brain, skin, heart, liver, and kidneys in proportions that vary by tissue, age, and pathological processes (1).

Macrophages are a major source of inflammatory cytokines (e.g., TNFα, IL6, and IL1) implicated in the pathogenesis of inflammatory diseases such as rheumatoid arthritis (RA) and inflammatory bowel disease (IBD) (11, 12). Macrophages drive protective immunity in response to pathogenic insults, but similar responses mounted against innocuous dietary proteins or commensal bacteria can lead to the development of chronic inflammatory disorders such as celiac disease and Crohn's disease, respectively. Gene expression studies have shown that activated macrophages are associated with IBD pathogenesis; however, their exact role in these diseases has not yet been elucidated (12). In disease states, the functions of various macrophages can be very different. Infiltrating monocytes are recruited from blood vessels and produce inflammatory mediators important for disease progression, but do not persist after the resolution of inflammation. In contrast, activated resident microglia proliferate locally, persist, and return to quiescence following remission (13). While KCs can be protective in disease states such as drug-induced liver injury and toxin-induced fibrosis, their functional dysregulation can contribute to chronic inflammation in the liver, including alcoholic, and non-alcoholic fatty liver diseases (NAFLDs/NASH) (14). In neurodegeneration models, microglial cells contribute to neuronal damage during disease development (15). Thus, tissue-resident or infiltrating monocytes can acquire either pro- or anti-inflammatory phenotypes. In cancer, tumor-associated macrophages (TAMs) and monocytes can promote immune-suppressive microenvironments to counteract immune evasion (16, 17). By secreting cytokines such as colony-stimulating factor 1 (CSF1), tumors are able to recruit macrophages and support tumorigenesis by enhancing angiogenesis and metastases *via* the secretion of metalloproteinases and inhibiting antitumor immunity by secreting immunosuppressive cytokines, such as IL10 (10–12). Both CSF1 and IL34 are expressed in multiple tumors (18, 19). The role of IL34 for tumor macrophages is unclear.

CSF1R signaling *via* CSF1 and/or IL34 ligands regulates the production and differentiation of most circulating and tissue-resident macrophages. CSF1R is expressed in multiple tissues including cerebral cortex, thyroid, lung, spleen, and liver, and more specifically, expressed in multiple cell subsets such as hematopoietic stem cells, monocytes, macrophages, microglia, LCs, and osteoclasts to regulate their development (20, 21). IL34 and CSF1 can bind and activate CSF1R, but their distinct expression has evolved to regulate systemic or local cellular differentiation. CSF1 is systemically expressed whereas IL34 is selectively expressed in the skin and central nervous system (19, 21). CSF1R knockout mice have significantly reduced macrophages, microglia, LCs, and osteoclasts. CSF1 spontaneous null mutation mice (CSF1^OP/OP^, Osteopetrotic), on the other hand, do not completely recapitulate the phenotype of CSF1R^−/−^ mice (22–25). CSF1^OP/OP^ mice have only a modest reduction in microglia but maintain normal LC development, exhibiting delayed macrophage development, and osteoclastogenesis (24). The discovery of IL34 and its role in microglia and LCs provided an explanation for the differences observed between CSF1R^−/−^ and CSF1^OP/OP^ mice. Although both IL34 and CSF1 bind to CSF1R, IL34 binds with higher affinity and thus can outcompete CSF1 binding to CSF1R (26, 27). IL34 also interacts with receptor protein tyrosine phosphatase-z, which is co-expressed with the CSF1R on neural progenitor cells (28). Based on the *in vitro* studies, IL34 and CSF1 may differentially potentiate macrophage differentiation where IL34 can drive IL10 and CCL17 (29).

Studies in CSF1R^−/−^, CSF1^OP/OP^, and IL34^−/−^ mice have illuminated the compensatory and unique functions of IL34 or CSF1 in development. However, the exact role of CSF1 and/or IL34 in adult mice independent of their roles in development has not been clarified. Our studies with CSF1 and/or IL34 neutralizing antibodies reveal novel insights into how these cytokines impact macrophage differentiation at steady state and disease using preclinical models in adult mice.

## Materials and Methods

### CIA and TNFΔ^ARE^ Arthritis

All animals used were purchased from The Jackson Laboratory or bred at Genentech. C57BL/6 and BALB/c mice were purchased from The Jackson Laboratory. Male DBA/1J mice 8–9 weeks old were obtained from The Jackson Laboratory and maintained in accordance with American Association of Laboratory Animal Care guidelines. All animal experiments were approved by the Genentech Institutional Animal Care and Use Committee (IACUC). Antibodies used *in vivo* were administered by intraperitoneal (i.p.) injection in phosphate buffered saline (PBS). Animals were dosed 3 times per week, 200 μg per antibody per dose for 1, 2, or 4 weeks. At the end of the study, animals were euthanized and spleen, brain, intestine, liver, bone marrow, lung, and skin were collected and fixed in formalin for pathology and immunohistochemistry (IHC) analysis. Fresh cells from part of spleen were collected for FACS.

DBA/1J mice were immunized i.d. at the base of the tail with 100 μg of bovine collagen type II (CII, Chondrex) emulsified in 100 μl of CFA on day 0. On day 21, mice received a booster injection of 100 μg of CII in 100 μl of incomplete Freund's adjuvant. To assess efficacy, mice were randomly divided into different groups at day 24 post-initial immunization and were treated for 7 weeks. In a therapeutic model, disease mice at day 31 were randomized based on clinical arthritis scores and treated i.p. with aIL34 and aCSF1, alone or in combination, TNFRII-Fc or anti-Ragweed (aRW). All treatments were administered at 200 μg/mouse in 100 μl of saline 3 times per week for 7 weeks, except TNFRII-Fc, which was given at 150 μg/mouse 3 times per week. Mice were examined weekly for clinical signs of joint inflammation in each paw.

Mice were examined for signs of joint inflammation starting 1 week post immunization (mouse CIA) or starting at 6–8 weeks of the age (TNFΔ^ARE^). The severity of disease in each paw was graded on a scale of 0–4, according to an in-house scoring system. A score of 0 was assigned for normal joint appearance and a score of 1 was assigned cumulatively to each paw for erythema and/or edema in tarsal or carpal joints, metatarsal or metacarpal joints, metatarsalphalangeal (MTP) or metacarpalphalangeal (MCP) joints, or phalanges. A maximal score of 4 indicated erythema, edema, or both involving the entire paw. The maximal disease index for each mouse was 16.

### DSS Colitis

Acute colitis was induced by administration of 3% DSS (w/v, molecular mass 36–50 kDa; MP Biomedicals) in drinking water *ad libitum* as described (30). DSS was given for a total of 6 days (day 0 through 5), after which animals were given regular drinking water from day 6 through 8. Animals were weighed daily starting at day 4 of DSS administration until day 8. On day 8, animals euthanized by cardiac puncture under anesthesia and colons and mesenteric lymph nodes (LNs) were removed and analyzed. The treatment started 1 day before DSS treatment. Two hundred micrograms of antibody per dose in 100 μl of PBS was injected i.p. twice per week.

### TNFΔ^ARE^ Ileitis

TNFΔ^ARE^ mice were bred as TNFΔ^ARE^/WT heterozygous as previously described (31). Arthritis was scored from 6 to 19 weeks of age on a weekly basis. At 19 weeks of age, animals were randomized into groups based on the average clinical score and body weight. Treatment with neutralizing antibodies began at 20 weeks of age and was administered 3 times per week, at 200 μg per antibody per dose (400 μg of total antibody and 400 μg for isotype control). During the study, body weight was recorded weekly and arthritis scores were recorded at least 2 times per week. After 8 weeks of treatment, animals were euthanized and tissues were processed as described below.

Small intestine or colons were prepared as a “Swiss roll,” and fixed in formalin. Tissues were embedded into paraffin blocks and 5-μm sections were prepared. Slides were stained with H&E. For DSS, slides were scored by one of two experienced pathologists in four anatomical regions of the colon: the proximal colon (PC), middle colon (MC), distal colon (DC), and rectum (R). Each region was given a raw score based on crypt epithelial cell loss with consideration of the extent of inflammatory cell infiltrate, on a scale from 0 (healthy) to 5 (severe diffuse colitis characterized by complete loss of colonic epithelial cells). The raw scores from each region were summed to give total raw colitis severity score for each animal, which ranged from 0 (least severe) to 20 (most severe).

For TNFΔ^ARE^, small intestines were scored. Enteritis lesions in TNFΔ^ARE^ mice were scored on a combination of severity and extent of small intestinal involvement. The entire small intestine from proximal duodenum to the ileocecal junction was collected, flushed with saline, and fixed in 10% buffered formalin in a “jelly roll” configuration. Paraffin-embedded tissues were sectioned at 5-μm thickness and stained with hematoxylin and eosin (H&E). Sections were scored on the extent of inflammatory infiltrate in the lamina propria (minimal, mild, moderate, or severe), on extension of inflammatory infiltrate to submucosal and muscular layers (transmural involvement), and on regional extent of inflammation (involvement of proximal small intestine vs. ileum only). Scoring criteria were as follows: 1 = minimal inflammation of ileum; 2 = mild inflammation of ileum; 3 = mild/moderate inflammation of ileum; 4 = moderate/severe inflammation of ileum with evidence of transmural involvement; 5 = severe transmural inflammation of ileum or significant involvement (mild/moderate or greater) of proximal small intestine with moderate or greater inflammation of ileum.

### IL10 Knockout Spontaneous Colitis

IL10-null colitis was performed as previously described (32). Five- to 6-week-old IL10 null mice were fed with 200 ppm piroxicam containing mouse powder diet for 12 days. After an additional 4 weeks of normal mouse chow feeding, IL10 KO mice were treated with different antibodies at 10 mg/kg 3 times a week for 6 weeks.

### Accelerated NZB × NZW F1 Lupus

Accelerated NZB × NZW F1 lupus model was done as previously described (33). Adenovirus-5 (Ad5)-IFN-α or Ad5-LacZ control viral vectors, 2 × 10^8^ pfu, were administered by intravenous injection into 12-week-old female mice. Three weeks post Ad5 viral vector injection aCSF1 or aIL34 or in combination was dosed at 10 mg/kg subcutaneously twice per week for 8 weeks. Cyclophosphamide (CYC; Baxter) was used as a reference treatment and dosed at 30 mg/kg i.p. every 10 days. Proteinuria was assessed biweekly by colorimetric measurement using dipstick Multistix 10 SG (Bayer Diagnostics). Measured protein concentrations were categorized as trace, ≤ 30/dl, ≤ 100, and ≥300 mg/dl. Mice were considered to have severe proteinuria if two consecutive urine samples had a protein concentration of ≥300 mg/dl.

### MC38 Tumor Model

Female C57Bl/6 mice were subcutaneously inoculated with 1 × 10^5^ MC38 tumor cells in HBSS supplemented with matrigel in the right hind flank. Mice were recruited into groups that displayed average tumor volumes of ~150 mm^3^. Mice were administered 20 mg/kg of isotype control antibody or 10 mg/kg anti-M-CSF and/or 10 mg/kg anti-IL34 antibody intraperitoneally 3 times per week. Mice that received anti-M-CSF or anti-IL34 alone were also administered 10 mg/kg of isotype control antibody, so all treatment groups received 20 mg/kg of total antibody on treatment days. Tumor volumes were measured 3 times a week for the duration of the studies. The MC38 tumor model is a relatively fast-growing model and mice within our facility are required to be euthanized when tumors reach 2,000 mm^3^. In addition, this tumor model is prone to ulcerations that become more prevalent with time. Mice exhibiting ulcerations are required to be taken down due to our animal facility guidelines. Mice were removed from study once tumor volumes reached 1,500 mm^3^ and were considered fatalities due to tumor burden. Mice that exhibited ulcerations prior to achieving tumor volumes of 1,500 mm^3^ were removed from study and not included for tumor growth and survival analysis. For cellular analysis, mice were euthanized 12 days following the initiation of treatment, and tumors were harvested, weighed, and coarsely minced. Tumors were placed in CDTI buffer (high glucose DMEM, 10 mM HEPES, 5% fetal bovine calf serum, 2 mg/ml Collagenase D, 40 U/ml DNAse, 1 mg/ml trypsin inhibitor) and partially dissociated using gentleMACS C tubes on m_impTumor_03 setting using the gentleMACS Dissociator (Miltenyi Biotec). Samples were placed at 37°C for 45 min at 180 rpm. Single cell suspension was generated with gentleMACS Dissociator on m_impTumor_01 setting. Red blood cells were lysed, samples were strained through a 100-μm filter and resuspended in FACS buffer (PBS, 1% BSA, 0.01% NaN_3_). For *ex vivo* T cell stimulation, 3 × 10^6^ cells were plated in full media supplemented with 10% T cell stimulation medium (BD Biosciences), Brefeldin A (BD Biosciences), as well as PMA and ionomycin (eBiosciences) and incubated for 5 h at 37°C. Non-viable cells were identified by exposure to Fixable Viability Dye eFluor 780^®^ according to the manufacturer's protocol (eBioscience). To prevent Fc receptor binding of antibodies, cells were incubated with unconjugated anti-CD16/32 antibody at 4°C for at least 10 min. Fluorochrome-conjugated antibodies were used to stain the surface of cells (anti-CD45, clone 30-F11; anti-CD11b, clone M1/70; anti-F480, clone BM8; anti-Gr1, clone RB6-8C5; anti-CD11c, clone N418; anti-CD3, clone 17A2; anti-CD4, clone RM4-5; anti-CD8a, clone 53-6.7; anti-NK1.1, clone PK136; anti-CD86, clone GL-1; anti-I-A/I-E, clone M5/114.15.2; anti-PD-L1, clone 10F.9G2). For intracellular staining, cells were fixed, permeabilized, and stained with fluorescently labeled antibodies (anti-Ki67, clone SolA15; anti-NOS2, clone CXNFT; anti-Arginase 1, R&D Systems catalog number IC5868A; anti-FoxP3, clone FJK-16s; anti-Granzyme B, Molecular Probes catalog number MHGB04, anti-IFN-γ-alone XMG1.2; TNF-α, MP6-XT22) using the FoxP3 Transcription Factor Fixation/Permeabilization Buffer (eBioscience) according to the manufacturer's instructions. Samples were analyzed using an LSRII flow cytometer (BD Biosciences), and data were evaluated using FlowJo software (Tree Star). Cell numbers were quantified by multiplying the fraction of specific cell population fraction by the total number of cells isolated from the tumor.

### Toxicology Studies

In a repeat dose study, 8- to 10-week-old B6C3F1 female mice (*n* = 5/group) were dosed i.p. twice weekly for 7, 14, or 28 days with 400 μg/mouse of isotype control (aRW) or aCSF1/aIL34 antibodies. On study days 7, 14, or 28, animals were euthanized and serum was collected for liver biomarker analysis, plasma was collected for miR-122 analysis, and liver tissues were processed for ICH analysis of KC number. To evaluate effects of APAP, B6C3F1 female mice (*n* = 5/group) were dosed with 1,200 mg/kg of APAP or vehicle control *via* oral gavage. Six hours following dosing, animals were euthanized and serum was collected for liver biomarker analysis, plasma was collected for miR-122 analysis, and liver tissues were processed for anatomic pathology analysis.

### Analysis of Small and Large Intestines

In organ cultures, small intestine or colons were flushed with cold RPM1 1640 supplemented with 100 mg/ml penicillin/streptomycin. For colon, the entire colon (from rectum to cecum) was removed. For small intestine, the ileum (approximately the first 5 cm upstream of the cecum) was removed. Tissues were cut longitudinally and cultured in 12-well plates containing 1 ml of RPMI 1640 supplemented with 100 mg/ml penicillin/streptomycin for 24 h at 37°C. Supernatants were collected and centrifuged at 14,000 rpm for 5 min at 4°C. Colon lysate was processed by snap-freezing a piece of colon in LN2. Frozen tissue was stored at −80°C until processing. Add 0.5 ml 1 × lysis buffer to a 2-ml Eppendorf tube, and keep it on ice. Place tissue (about 100 mg) in lyses buffer. Add 5 mm bead (Qiagen Stainless Steel Beads 5 mm) and shake it at 30 Hz for 12 min at 4°C. Incubate it on ice for 20 min. Spin down at 14,000 rpm for 10 min at 4°C. Collect supernatant into a 1.5-ml Eppendorf tube. Store the protein solution at −80°C until analysis.

### IHC and Digital Image Analysis

Intestines, liver, skin, and brain were harvested and fixed in 10% neutral buffered formalin for 24 h and then processed for paraffin-embedded histology using routine methods. Four-micron histologic sections were stained using a Ventana XT Discovery system with 1 μg/ml rat anti-mouse F4/80 mAb C1:A3-1 (Serotec), 5 μg/ml rabbit anti-langerin IgG (Novus Biologicals), or 0.25 μg/ml rabbit anti-Iba-1 (Wako Chemicals) as indicated. Secondary detection was with OmniMAP-HRP (Ventana) for F4/80 and Iba-1 and DABMap (Ventana) for langerin. Whole-slide digital imaging was performed on an Olympus Nanozoomer and images were imported into MATLAB software, version 7.14 (MathWorks), for morphometric quantitation of DAB-positive tissue area using intensity and color thresholding. Total tissue area of interest was defined automatically using standard morphologic features, except in hippocampus regions, which were defined manually. For data plotting, DAB-positive tissue area was normalized to the total tissue area analyzed.

### Joint Cortical Bone Volume (JCBV) and Cartilage Micro-Computed Tomography (μCT)

The μCT imaging and image analysis technique used to quantify cortical bone destruction has been described in detail previously (34) and is briefly described here. Limbs were excised above the ankle and fixed in formalin in preparation for the μCT imaging, which was performed on an *ex vivo* μCT scanner (μCT 40; Scanco Medical, Brüttisellen, Switzerland) at 16 μm isotropic voxel size, 1,000 projections per full rotation, and an integration time of 300 ms. The x-ray tube was operated at 45 kVp voltage and 177 μA current. An automated image analysis technique was used to locate and quantify the cortical bone volume within close proximity of 5 metatarsophalangeal and 3 metacarpophalangeal joints. The combined volume for all four paws per animal, JCBV, is a sensitive metric to cortical bone destruction at the joints.

For the evaluation of cartilage loss using μCT, the formalin-fixed mice hind limb paws were processed for contrast-enhanced cartilage imaging. The iodinated contrast agent used was the non-ionic aqueous Isovue-370 (Iopamidol 76% from Bracco Diagnostics, Monroe Township, New Jersey). The specimens were partially skinned and the toe tips were snipped using a surgical blade to improve the access of the contrast agent. Specimens were then soaked in polypropylene tubes containing 2 ml of 30% Isovue in PBS for 48 h. The specimen tubes were placed on a rocker (Nutator from Becton Dickinson, Franklin Lakes, New Jersey) during this process to induce a uniform distribution of the contrast agent. All the specimens were then scanned in an *ex vivo* μCT scanner (μCT 50; Scanco Medical, Brüttisellen, Switzerland) at 5 μm isotropic voxel size with a 1,500-ms integration time, two signal averages, and 2,000 projections per rotation. The x-ray tube was operated at 45 kVp and 133 μA current. Soybean oil was used as a scanning medium to avoid the diffusion of aqueous contrast agent out from the specimen.

For image analysis, all the tomographic images were loaded in Analyze 3D viewing software (AnalyzeDirect, Inc.), and the intensities were uniformly windowed from −800 to 28,600 (image intensity units from Scanco system) for visual scoring. Since cartilage does not take up significant amounts of iodinated contrast agent during this procedure, the articular non-calcified cartilage appears as a low-intensity gap in the μCT images (35). The thick dark line at the metatarsophalangeal (MTP) joints was confirmed to be non-mineralized cartilage by histological analysis, and the amount of cartilage was assessed with a semi-quantitative scoring system. The cartilage scoring system employed a score range from 0 to 4 with the following scale: 0—thick continuous; 1—thick discontinuous, 2—thin continuous, 3—thin discontinuous, 4—no cartilage. All the five MTP joints were individually scored and averaged to get the total score per paw. The total cartilage score per mouse was obtained by averaging the total scores of both the hind limb paws. The cartilage imaging technique was shown to be well-correlated with histological analysis (*r* = 0.88, *p* < 0.001, Supplementary Figure 4).

### Analysis of Arthritis Joints

To assess the MOA of treatments on cellular infiltration in the ankle joints, mice with clinical arthritis score of 4 in each hind paw were randomized and received anti-IL34/anti-CSF1 combination or isotype control treatment. At day 14 post treatment, synovia from ankle joints was harvested and processed by collagenase digest to release the single cells for FACS analysis. The synovia from three mice in the same group was combined in order to get enough cells for FACS.

### Flow Cytometry and Antibodies

The cells were isolated from peripheral blood or tissues were analyzed by flow cytometry. Fluorescence FITC, PE, PerCP, Texas-Red, APC, APC-Cy7, PE-Cy7, and Pacific Blue-conjugated anti-mouse antibodies CD11b, CD11c, Ly6C, Ly6G, F4/80, CD45, CD4, CD8, and CD44 were purchased from BD Biosciences. Fluorescence-conjugated anti-mouse CSF1R was from eBioscience, and anti-mouse CCR2 was from R&D Biosystem. FACS was conducted on a flow cytometer (LSRII, BD Biosciences). Monocytes and macrophage subsets were identified as described in figure legends. Statistical analysis was performed using ANOVA with Dunnett's test.

### Neutralizing Murine CSF1 and IL34 Antibodies and PK Assays

Monoclonal blocking antibody rat anti-mouse CSF1 (mIgG2a) were generated in-house. The affinity (*K*_d_) of aCSF1 to mouse CSF1 is about 9.3 nM by biacore analysis. Phage derived anti-mouse IL34 blocking mAb were generated in Genentech with 21.3 nM affinity biacore. Using monocyte differentiation *in vitro* assay, the antibodies were able to block soluble cytokine (50 ng/ml)-induced cell differentiation effectively (50% maximum inhibitory concentration (IC50) for aIL34 = 30 ng/ml; aCSF1 = 1.1 μg/ml).

For measuring drug levels of aIL34 in serum from dosed mice, ELISA plates were coated with 1 mg/ml muIL-34-his in PBS. Dilutions of affinity-matured, phage-derived aIL34 standards, controls, and samples were incubated on coated and blocked plates, detected with 0.5 mg/ml biotin-labeled rat anti-muIgG2a (BD Biosciences, San Jose, CA), followed by SA-HRP and TMB steps. Plates were read and analyzed as above. The assay detection range is 20–0.08 ng/ml and the lower limit of quantitation (LLOQ) is 8 ng/ml for murine serum samples with an initial dilution of 1:100.

For measuring drug levels of aCSF1 in serum from dosed murine disease models, ELISA plates were coated with 0.5 mg/ml murine CSF1 (R&D 416-ML/CF) in PBS. Dilutions of chimeric rat aCSF1 standards, controls (GNE), and samples were incubated on coated and blocked plates, detected with 0.2 mg/ml biotin-labeled rat anti-muIgG2a (BD Biosciences, San Jose, CA), followed by SA-HRP and TMB steps. Plates were read and analyzed as above. The assay detection range is 1,000–4 pg/ml and the LLOQ is 0.4 ng/ml for murine serum samples with an initial dilution of 1:100.

The serum concentration of aIL34 and aCSF1 vs. time data from each animal was analyzed using Phoenix WinNonlin version 6.4 (model: plasma 200–202) (Pharsight corporation, Mountain View, CA).

### Cytokine Analysis

To measure cytokines, mouse serum and colon tissues were collected. The colon lysate was made as described above. Analysis was performed by using affymetrix Panomix magnetic bead multiplex cytokine kits (Santa Clara, CA) and read on a Luminex reader, according to manufacturer's instructions. For human and mouse CSF1 measurements, ELISA was performed by using R&D Quantikine kits [human (DMC008), mouse (MMC00)] according to the manufacturer's instructions. For measuring human IL34 in patient and normal donor serum samples, microtiter 384-well Maxisorp plates were coated overnight at 4°C with 4 μg/ml anti-huIL34 (R&D Mab5265) in 25 μl/well PBS and blocked with block buffer (BB) (0.5% BSA in PBS). Plates were washed six times with 300 μl of wash buffer (WB; PBS, 0.05% Tween-20) between each step. Dilutions of human IL34-FLAG standards, controls (GNE), and test serum samples were prepared in high salt assay diluent (HSAD; 0.5% BSA, 10 ppm Proclin, 0.05% Tween-20, 0.2% bovine γ-globulin, 0.25% CHAPS, 5 mM EDTA, and 0.35 M sodium chloride in PBS, pH 7.4). A heterophilic antibody blocker, Immunoglobulin Inhibiting Reagent (IIR; Sera Lab, West Sussex), was added at 1 mg/ml to serum samples prior to dilution. Twenty-five microliters of standards, controls, and samples were incubated on coated and blocked plates, detected with 0.25 μg/ml biotin-labeled hamster anti-IL34 (GNE) diluted in assay diluent (AD; PBS, pH 7.4, 0.5% BSA, 0.05% Tween-20, 10 ppm Proclin), followed by streptavidin-peroxidase (SA-HRP, Amersham Biosciences, Piscataway, NJ, RPN 4401) diluted 1:10,000 in AD. Color was developed using tetramethyl benzidine (TMB) (Moss, Pasadena, MD). Enzymatic detection reactions were stopped with 1 M H_3_PO_4_ and read on a microplate reader (BioTek, Winooski, VT) at 450 nm with a 650 nm reference. IL34 concentrations were interpolated from a four-parameter fit of the standard curve on each plate using software developed at Genentech. The assay detection range is 100–2 pg/ml and the LLOQ is 6 pg/ml for human serum samples with an initial dilution of 1:3. Up to 1 μg/ml of human soluble CSF1R did not interfere with detection of IL34 in the assay.

For measuring mouse IL34 in serum and tissue lysates from murine disease models, ELISA plates were coated with 2 μg/ml sheep anti-muIL34 (R&D AF5195) in coat buffer (CB) (0.05 M bicarbonate buffer, pH 9.6). Dilutions of mouse IL34 standards, controls (R&D 5195-ML-010), and samples (IIR was not added to mouse samples) were incubated on coated and blocked plates, detected with 0.125 μg/ml biotin-labeled sheep anti-muIL34 (R&D AF5195), followed by SA-HRP and TMB steps. Plates were read and analyzed as above. The assay detection range is 1,000–4 pg/ml and the LLOQ is 12 pg/ml for murine serum samples with an initial dilution of 1:3. Up to 1 μg/ml of murine soluble CSF1R and up to 1 mg/ml anti-IL34 did not interfere with detection of IL34 in the assay. For analysis of IL34/CSF3 expression in tumor lines, 1 × 10^6^ MC38, JC, CT26, EMT6, TC1, and 4T1 cells (ATCC) were plated into a single well of a six-well plate in 2 ml of media. Cells were cultured for 72 h and media was harvested and clarified by centrifugation. Fifty microliters of media was used to perform ELISAs for mouse M-CSF and IL34 (R&D Systems).

### Liver Injury Biomarkers and Liver Anatomic Pathology

Whole blood was collected at necropsy and processed to serum or plasma. ALT, AST, SDH, and GLDH were measured in serum samples using standard assays on an automated clinical chemistry analyzer and assay reagents as per manufacturer's recommendations. miR-122 was measured from plasma samples. Briefly, total RNA was extracted from plasma samples using the miRNeasy mini Kit (Qiagen, Valencia, CA); samples were processed according to the manufacturer's instructions. cDNA was generated with reagents from the bulk version of the High Capacity cDNA Reverse Transcriptase Kit (4387406 Life Technologies, Carlsbad, CA) or the High Capacity RNA-to-cDNA Kit (Life Technologies, Carlsbad, CA). cDNA was pre-amplified and real-time PCR was performed using the FluidigmTM Biomark high-throughput qPCR instrument (South San Francisco, CA) with commercially available reagents and kits with minor modifications. miR-122 expression was normalized against an average of four miRNAs (hsamiR-146a, hsa-miR-301, hsa-miR339-5p, and rno-miR-664). Liver tissues from APAP and vehicle control-treated mice were fixed in 10% neutral buffered formalin, paraffin-embedded, and H&E stained for routine histopathologic evaluation.

### Statistical Analysis

GraphPad Prism Version 7 (GraphPad, La Jolla, CA, USA) and JMP 9.0 were used for statistical analysis. Non-parametric *t-*test with unpaired two-tailed option was used for experiments containing two groups of analysis; one- or two-way ANOVA was used and compared with Dunnett's test for experiments containing more than two groups. *p* value < 0.05 was considered significant. ^*^p < 0.05, ^**^p < 0.01, ^***^p < 0.001, ^****^p < 0.0001.

## Results

### Requirement of CSF1 and/or IL34 During Tissue Macrophage Homeostasis

**Figure 1:**
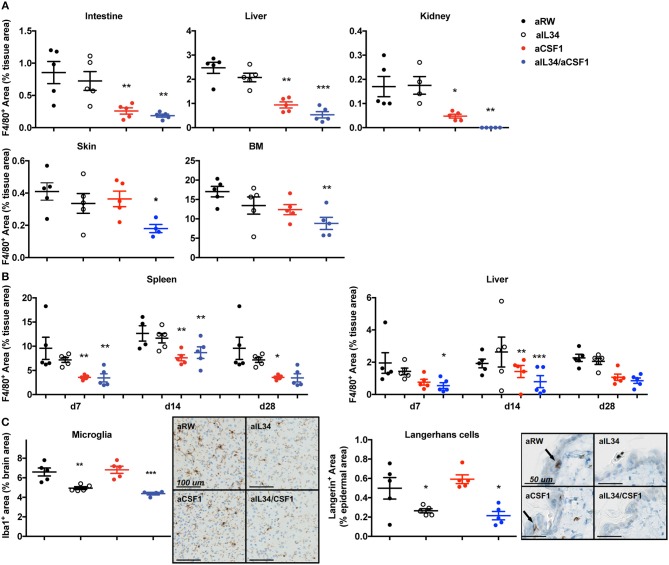
CSF1 and IL34 regulate macrophage homeostasis in adult mice. **(A)** Effect of aCSF1 and/or IL34 antagonist antibodies on tissue-resident macrophages. C57/BL6 mice were treated with aCSF1 and/or aIL34, or anti-ragweed (aRW) IgG2a intraperitoneally for 1 month. Formalin-fixed tissues (intestine, liver, kidney, skin, and bone marrow) were stained with F4/80 and stained area was plotted and percentage of total tissue area to quantitate tissue-resident macrophages. **(B)** Quantitation of tissue-resident macrophages in spleen and liver after 7, 14, and 28 days of aCSF1 and/or aIL34 treatment stained with F4/80. **(C)** Staining of tissue-resident brain microglia (stained with anti-Iba-1) or skin epidermal Langerhans cells (anti-Langerin). Immuno-reactive cells were quantitated by digital image analysis as described in Materials and Methods. Results are displayed as group means ± SEM (standard error of the mean), and are representative of at least two independent experiments. ^*^p < 0.05, ^**^p < 0.01, ^***^p < 0.001.

In order to elucidate CSF1- and IL34-mediated regulation of macrophage homeostasis, highly selective antibodies against CSF1 or IL34 were generated and shown to be biologically active *in vitro* (Supplementary Figure 1A). C57BL6 mice were treated with aCSF1 or aIL34, separately or in combination for 4 weeks followed by an evaluation of various tissues by IHC using F4/80^+^ to mark resident macrophages (Figure 1). Analysis of serum aCSF1 and aIL34 showed presence of saturating concentrations of both antibodies reaching 10 μg/ml after 1 month with a single dose treatment (Supplementary Figure 1B). IHC, FACS, and reporter mice were used to quantitate tissue macrophages. IHC analyses accurately quantitate macrophages for a number of tissues (e.g., intestine, liver, kidney, skin, spleen) where tissue processing and digestion complicates reliable *in silico* analysis of resident macrophages. IHC readouts are absolute quantitations of IHC+ signal in each of these tissues, expressed as the cumulative area of IHC+ cells per μm^2^ of tissue area analyzed. In the intestine, liver, kidney, bone marrow, and spleen, the number of resident macrophages declined in mice treated with aCSF1 or aCSF1/aIL34; however, treatment with aIL34 alone did not impact F4/80^+^ cells (Figures 1A,B). In skin, subepithelial macrophages declined in mice treated with aCSF1/aIL34. Surprisingly, kidney macrophages were nearly absent in animals receiving both aIL34 and aCSF1 (Figure 1A). Kinetic studies revealed that the depletion of resident macrophages in the spleen and/or KCs in the liver occurs within 7 days post treatment (Figure 1B).

To better dissect macrophage subsets affected by CSF1 and IL34, we utilized CX_3_CR1^wt/gfp^ mice as previously described (26, 36–38). CD11b^+^ hematopoietic cells can be divided into three subsets in CX_3_CR1^wt/gfp^ mice: CX_3_CR1^negative^, CX_3_CR1^intermediate^, and CX_3_CR1^high^. CD11b^+^CX_3_CR1^negative^ includes various leukocytes such as eosinophils and neutrophils depending on the tissue. The CD11b^+^CX_3_CR1^int^ cells express F4/80 and MHCII but low levels of Ly6C and CCR2, representing tissue-resident macrophages (26). The CD11b^+^CX_3_CR1^hi^Ly6C^hi^CCR2^+^ cells represent inflammatory monocytes or macrophages. CX_3_CR1^wt/gfp^ mice were treated with aCSF1 and aIL34, separately or in combination for 4 weeks and, subsequently, were evaluated for their tissue-resident macrophage composition. Analyses of monocytes in the blood or spleen suggested that circulating monocytes depend on CSF1 (Supplementary Figures 2A,B). In agreement with IHC studies, dual IL34 and CSF1 blockade reduced kidney macrophages (Supplementary Figure 2C). In the colon, the CX_3_CR1^high^ and CX_3_CR1^intermediate^ subsets were respectively highly and partially dependent on CSF1 (Supplementary Figure 2D).

After 1 month of aIL34 treatment, the number of LCs in the skin epidermis and microglia found in the gray matter of the brain declined in mice, whereas these populations were not affected by aCSF1 treatment (Figure 1C). However, the lack of brain penetrance (via the blood–brain barrier) may explain the subtle effect of aIL34 on microglia. Our data suggest that various tissue macrophages have distinct requirements for CSF1 and/or IL34. IL34 plays an important role in the maintenance and differentiation of LCs and microglia in the gray matter of adult mice, while CSF1 plays a dominant role in macrophage homeostasis in other tissues such as colon and liver. Certain macrophages localized to the kidney, depending on both IL34 and CSF1. Thus, therapeutics aimed at CSF1 and/or IL34 may have uniquely impacted different subsets of tissue macrophages.

### IL34 and CSF1 Expression in Inflammatory Diseases

To understand whether IL34 and/or CSF1 have pathogenic roles in disease, we surveyed the expression of both in RA, osteoarthrosis (OA), and IBD. IL34 was elevated in the serum and synovial fluid from RA donors, compared to OA donors (Supplementary Figure 3A). CSF1 was elevated in both RA and OA serum compared to healthy donors, while in the synovial fluid, CSF1 was increased only in RA donors likely due to the abundance of macrophages and fibroblasts (Supplementary Figure 3A) (11). In a mouse model of collagen-induced arthritis (CIA), serum IL34 (but not CSF1) was elevated compared to normal mice (Supplementary Figure 3B).

Both IL34 and CSF1 transcripts were elevated in inflamed gut tissues from Crohn's disease (CD) or ulcerative colitis (UC) patients compared to non-IBD control diverticulitis and normal tissues that were tumor-adjacent (Supplementary Figure 3C). CSF1R message was not significantly altered; however, CCR2 and CCL2 involved in macrophage recruitment (39) were both increased in both CD and UC (Supplementary Figure 3C). Both IL34 (only trended) and CSF1 were increased in mouse DSS-induced colitis (Supplementary Figure 3D). TNFΔ^ARE^ mice (31) are a model in which mice develop symptoms that phenocopy CD. Analyses of cultured gut tissues from TNFΔ^ARE^ mice showed that CSF1 was increased in the ileum and cecum, whereas IL34 was elevated primarily in the jejunum and ileum (Supplementary Figure 3E). While the expression studies do not identify a causal role or cellular source for any of these cytokines, it suggests that both cytokines may impact macrophage differentiation and inflammation in the gut or joints.

### Blockade of Both IL34 and CSF1 Attenuates Lesions in Murine Arthritis Models

**Figure 2:**
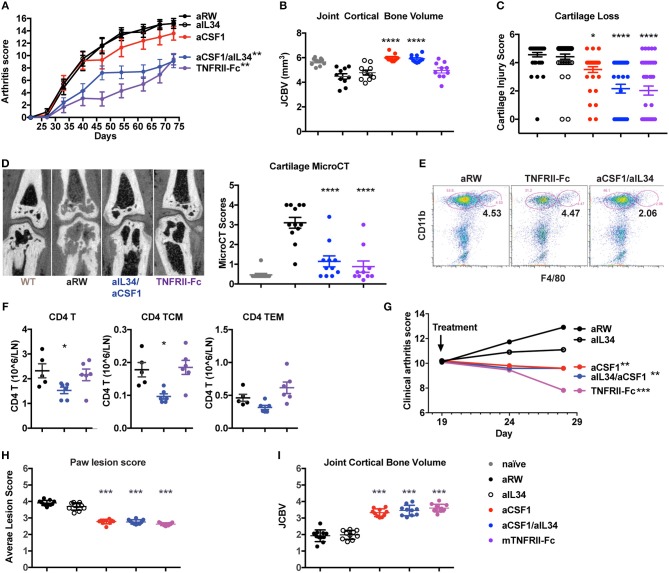
Reduced induced or spontaneous mouse arthritis treated with aCSF1 and/or IL34. **(A)** Reduced longitudinal clinical score in CIA. DBA/1J mice were randomized at day 24 and then treated with aIL34 and/or aCSF1 for 7 weeks. Murine TNFRII-Fc decoy was used as a standard of care control. Isotype control includes anti-ragweed (aRW). Mice were examined weekly for joint inflammation in each paw. **(B)** Joint cortical bone volume (JCBV) determined by micro-CT (μCT) was used to quantify bone remodeling at the base of metatarsal bones. One hundred percent bone volume was set for naive mice. Anti-RW control group had lowest JCBV (average 79%) due to the bone erosion. TNFRII-Fc improved it to 87% and aIL34 to 84%. aCSF1 or dual aCSF1/aIL34 maintained the bone volume at a healthy level (106 and 104%). **(C)** Cartilage injury histology score (*n* = 38–40/group). **(D)** Analysis of cartilage damage by contrast-enhanced μCT imaging. Representative micro-CT images shows cartilage as dark rim at joint surface (left set of images). The cartilage scoring system employed a score range from 0 to 4 with the following scale: 0—thick continuous, 1—thick discontinuous, 2—thin continuous, 3—thin discontinuous, 4—no cartilage. All the five MTP joints were individually scored and averaged to get the total score per paw. The total cartilage score per mouse was obtained by averaging the total scores of both the hind limb paws (*n* = 6 per group). Anti-CSF1 or anti-IL34 monotherapies were not tested in this study. **(E)** Joint inflammatory CD11b^+^F4/80^+^ macrophages are reduced with aCSF1/aIL34 treatment. Arthritic mice, with a disease score of 4, were randomized and received anti-IL34/anti-CSF1, aRW, or TNFRII-Fc for 7 days. Ankle joints were harvested and processed for FACS. Each data point represents samples pooled from three mice. **(F)** Reduced CD4 T central memory (TCM) and in draining lymph nodes. **(G)** TNFΔ^ARE^ spontaneous arthritis, anti-CSF1 significantly reduces clinical arthritis score. **(H)** TNFΔ^ARE^ arthritis paw lesion histology score is improved by aCSF1. **(I)** TNFΔ^ARE^ arthritis JCBV is improved with aCSF1. Data are displayed as mean ± SEM. ^*^p < 0.05, ^**^p < 0.01, ^***^p < 0.001, ^****^p < 0.0001.

Given that the expression of IL34 and CSF1 are both elevated in arthritis, and that CSF1R signaling is critical for macrophage differentiation and osteoclast homeostasis, we asked whether CSF1 and/or IL34 neutralization affected arthritis disease induction and outcome. To do this, we used two mouse models: CIA and TNFΔ^ARE^ spontaneous inflammatory polyarthritis [in which a deletion of the AU-rich element (ARE) results in increased TNF expression (31)] (Figure 2). Animals were treated with aIL34 or aCSF1 alone or in combination, and the TNF antagonist TNFRII-FC was used as a positive control to block disease progression. After 7 weeks of treatment, longitudinal arthritis clinical scores indicated that only dual blockade of CSF1 and IL34 or TNFRII-Fc treatments were protective in CIA (Figure 2A). In this model, joint inflammation resulted in bone remodeling, which was visualized and quantified by μCT as JCBV. The dual blockade of CSF1 and IL34 or CSF1 alone equally normalized JCBV (Figure 2B), while aIL34 alone had no effect. This finding was confirmed by histological evaluation of cartilage injury showing significant reduction of cartilage loss in the groups treated with TNFRII-Fc or aCSF1/aIL34 combination or aCSF1 alone compared to the isotype control or aIL34 treatment (Figure 2C). In a separate study, contrast-enhanced μCT imaging corroborated the above observations; dual blockade of IL34/CSF1 in a therapeutic design was equally effective as TNFRII-Fc in ameliorating cartilage injury, in line with histology (Figure 2D and Supplementary Figure 4). Interestingly, both aCSF1 and aCSF1/aIL34 combination therapy had an isolated effect on JCBV not seen with TNFα blockade (Figure 2B), suggesting yet an additional mechanism modulates osteoclast function. This is reminiscent of the therapeutic targeting of the RANKL/OPG pathway that regulates osteolysis vs. osteogenesis independent of the associated inflammation (40). This effect, however, was not observed in the TNFΔ^ARE^ study, in which TNFα blockade was equally efficient at restoring JCBV (Figure 2I), raising the possibility that the reference treatment in the first study had suboptimal efficacy resulting in the discrepancy seen.

Macrophages may bridge the innate and CD4 T cells during inflammatory response; thus, we evaluated the effect of aCSF1 and/or aIL34 treatment on cellularity in ankle joints and LNs of arthritic mice. Arthritic mice with a disease score of 4 were treated with the control aRW, TNFRII-Fc, or a combination of aCSF1/aIL34 for 1 week. aCSF1/aIL34 reduced inflamed tissue macrophages in the ankle joint (Figure 2E), whereas total CD4 T cells and CD4 T central memory cells were reduced in LNs (Figure 2F). Thus, dual treatment reduces macrophages as well as other pathogenic immune cells such as CD4^+^ T cells.

TNFΔ^ARE^ mice spontaneously develop arthritis by 16 weeks of age accompanied by macrophage influx in the hind paws (31) (Supplementary Figure 5). TNFΔ^ARE^ is a complex disease model reflective of several inflammatory pathways in addition to TNF (41). Blockade of CSF1 alone or of both CSF1/IL34 for 9 weeks reduced arthritis compared to treatment with the aRW control (Figure 2G) and histologically (Figure 2H), including a reduction of bone remodeling (Figure 2I). Our results demonstrated that combined blockade of IL34 and CSF1 was protective in preclinical models of arthritis, similar to the effect seen with treatment of a TNF antagonist supporting the notion that macrophages are at the center of an inflammatory cellular circuit in mouse models of arthritis (CIA and TNFΔ^ARE^). Our analyses further suggest that CSF1 positively regulates pathogenic macrophages and monocytes in these murine models of arthritis.

### Blockade of Both CSF1 and IL34 Is Protective in Murine Models of Colitis and Ileitis

**Figure 3:**
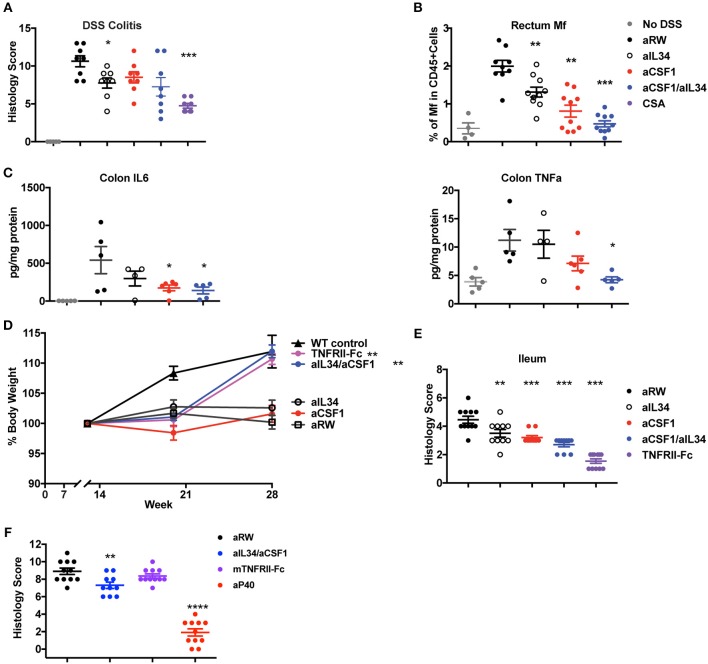
Reduced induced or spontaneous mouse colitis treated with aCSF1 and/or IL34. **(A)** Dual blockade of CSF1 and IL34 reduced DSS-induced colitis. Acute colitis was induced by administration of 3% DSS in drinking water at day 0 for total 6 days. Mice were treated with aCSF1 and/or IL34 1 day prior to DSS administration. Cyclosporine A (CSA) was used as a positive control treatment arm. Dual blockade of CSF1 and IL34 improved the disease (32%, ^*^p < 0.05) compared to aRW disease control. **(B)** Colon rectum cellularity: Single or dual blockade of CSF1 and IL34 reduces macrophages. Anti-CSF1 or aIL34 reduces macrophages compared to aRW isotype control group. However, dual blockade showed superior to single blockade with aIL34 alone. **(C)** Serum cytokines: Reduction of TNFα and IL6 by aCSF1 or aCSF1/aIL34 blockade. **(D)** TNFΔ^ARE^ ileitis: Improved body weight in TNFRII-Fc or aCSF1/IL34 combination treatment. **(E)** Reduced ileitis histology score with aIL34 and/or aCSF1 as well as TNFRII-Fc compared to control aRW group. Data are displayed as mean ± SEM. **(F)** Reduced colon histology score in IL10 null colitis model treated with aCSF1/IL34 compared to TNFRII-Fc. Positive control includes anti-p40. ^*^p < 0.05, ^**^p < 0.01, ^***^p < 0.001, ^****^p < 0.0001.

Macrophages and neutrophils comprise a hallmark cellular signature of TNF non-responders in IBD tissues (12). Elevated expression of IL34 and CSF1 in human IBD tissues and preclinical models prompted us to assess the effect of IL34 and/or CSF1 neutralization in murine IBD models. One day prior to DSS challenge, mice were treated with either aCSF1 or aIL34 alone, aCSF1/aIL34 or aRW isotype control, as well as the immune-suppressant, cyclosporine A (CSA). Dual blockade of CSF1 and IL34 was slightly more efficacious at preventing disease in DSS colitis than the monotherapies, reducing the histology score compared to the control treatment but was less efficacious than the treatment with CSA, which reduced the histology score by >50%. FACS analysis showed reduced numbers of CX_3_${\text{CR}}_{1}^{\text{high}/\text{int}}$ macrophages compared to isotype control antibody (Supplementary Figures 6, 7). Single treatment with either CSF1 or IL34 was only marginally beneficial (Figure 3A). Accordingly, in the inflamed rectum, combination of IL34/CSF1 treatment reduced gut macrophages to a greater extent than aIL34 or aCSF1 treatments alone (Figure 3B). Colon tissue cytokines such as TNFα were also reduced in aCSF1/aIL34-treated mice (Figure 3C). However, the reduction of IL6 was primarily driven by CSF1. aCSF1 or aCSF1/aIL34 treatment significantly reduced circulating monocytes, in the blood, mesenteric LNs, and spleen, suggesting that this effect is primarily driven by CSF1 (Supplementary Figure 7). Blockade of CSF1 alone or CSF1/IL34 reduced the number of inflammatory (CX_3_CR1^hi^) macrophages in the colon with a minor impact on residential (CX_3_CR1^int^) macrophages (Supplementary Figure 6). Therefore, both CSF1 and IL34 are important in DSS-induced inflammation; however, CSF1 plays a dominant role in both gut macrophage differentiation and epithelial injury (42).

In TNFΔ^ARE^ mice, increased TNFα expression leads to the gradual development of ileitis presenting with diarrhea and body weight loss (31). Tissue-resident F4/80^+^ macrophages are increased at 19 weeks in TNFΔ^ARE^ ileum (Supplementary Figure 8). aCSF1 or aIL34 monotherapies did not protect against weight loss in this chronic inflammatory model (Figure 3D). However, dual CSF1/IL34 blockade improved body weight similar to treatment with a TNF antagonist (Figure 3D). Analysis of ileum tissue histology showed reduced gut inflammation and immune-pathology mainly induced by aCSF1 (Figure 3E). IL10 knockout mice spontaneously develop colitis (32). Both monocytes and macrophages are shown to be an important cell type to sense inflammatory signals *via* Myd88 to precipitate the disease (43). aCSF1/IL34 significantly reduced histological tissue inflammation better than TNFRII-Fc reference treatment; however, anti-p40 was more efficacious in this disease model (Figure 3F). Altogether, analysis of all three intestinal inflammatory models (DSS, TNFΔ^ARE^ and IL10 null mice) is consistent with the pathologic role of macrophages in gut inflammation, which may be targeted by aCSF1 and aIL34 therapeutics.

### CSF1 and IL34 Blockade Does Not Reduce Immune-Pathology in NZB/W F1 Lupus Model

Inflammatory circuits in lupus include autoantibodies against self-antigens such as nucleic acids presented as immune complexes, which can be taken up by tissue-resident macrophages. This phenomenon is captured in NZB/WF1 mice, used as a preclinical model of lupus. Given that the dual blockade of CSF1 and IL34 reduced numbers of kidney macrophages, we examined the effects of CSF1 and IL34 treatment on lupus disease progression. Ectopic expression of IFNα or Pristane was used to further accelerate immune-complex pathology in NZB/W F1 female mice prior to the initiation of the therapeutic study (33). NZB/W mice in this accelerated lupus model were treated with aCSF1, aIL34, or aCSF1/IL34, or with Cytoxan or aRW as controls to examine whether inhibiting macrophage/monocyte differentiation impacts proteinuria or survival (Supplementary Figure 9A). In mice ectopically expressing IFNα, we found that Cytoxan reduced proteinuria and improved survival, however, aCSF1 and/or aIL34 did not have similar effects (Supplementary Figure 9A). In Pristane-accelerated lupus, we also did not observe reduction in proteinuria or enhanced survival with aCSF1/aIL34 combination treatment compared to Cytoxan (Supplementary Figure 9B). Pathways such as B cell-mediated autoantibody or antigen presentation may override the macrophage-mediated immune-pathology in this preclinical model (33).

### CSF1, but Not IL34, Is Required for TAM Accumulation and Immune Homeostasis

**Figure 4:**
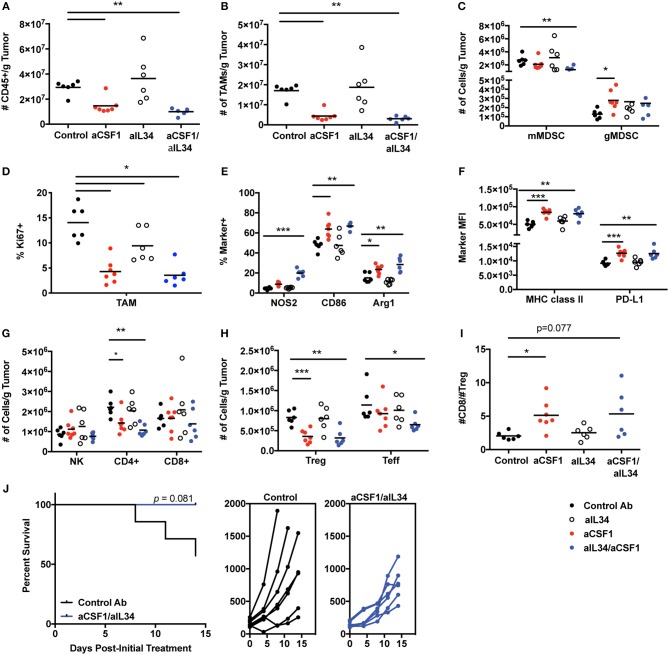
CSF1, but not IL34, is required for TAM and immune homeostasis within MC38 tumors. Female C57Bl/6 inoculated with MC38 tumors were treated with control, aCSF1, aIL34, or aCSF1/aIL34. MC38 tumors were harvested on day 12 post-initial treatment and the following parameters were evaluated: **(A)** total CD45^+^ cells; **(B)** TAMs per gram (g) tumor; **(C)** monocytic and granulocytic MDSC populations (mMDSC or gMDSC, respectively); **(D)** Ki67^+^TAMs; **(E)** functional marker analysis within TAMs including proinflammatory NOS2, CD86, as well as the anti-inflammatory marker arginase 1 (Arg1); **(F)** MHCII and PD-L1 TAM expression; **(G)** Number of NK, CD8, and CD4 T cells within tumors; **(H)** CD4^+^ Teff and Foxp3^+^ Tregs within tumors; **(I)** ratio of CD8 T cells to Tregs within tumors; and **(J)** survival of mice following control antibody aRW or aCSF1/aIL34 (left), and individual tumor growth curves (right). Functional and phenotypic data were derived from 6 to 7 mice per treatment group **(A**–**I)**. Each symbol indicates data from a single tumor harvested from an individual mouse. ^*^p < 0.05, ^**^p < 0.01, ^***^p < 0.001.

TAMs condition the tumor microenvironment by producing immunosuppressive factors to dampen anti-tumor effector cells and promote immune-suppressive cells such as Tregs (16, 17). Monocytes can also promote tumor metastasis and maintenance associated with poor prognosis; thus, therapeutic approaches targeted against monocyte recruitment and function may be beneficial against tumor growth (44). CSF1 and IL34 are expressed in various tumors (TCGA database) such as kidney cancers (Supplementary Figure 10). Several mouse tumor cell lines were screened for CSF1 and IL34 secretion *in vitro* in order to identify a relevant cell used for tumorigenesis and to test CSF1R ligand blockade *in vivo*. The MC38 tumor cell line expresses high levels of both cytokines, CSF1 (~900 pg/ml) and IL34 (~300 pg/ml) (Supplementary Figure 11), and thus was utilized to assess macrophage and immune homeostasis *in vivo*. In addition, TAMs are the most prevalent immune cell population detected within MC38 tumors (45) (Figure 4). The experiment was terminated when a sufficient number of mice remained for statistical analysis that were below the designated tumor volume size and that did not display ulcerations that were at 12 days post-treatment. *In vivo*, neutralization of CSF1 or CSF1/IL34 significantly reduced the total number of CD45^+^ immune cells within tumors when compared to control aRW-treated mice while no change was noted in aIL34 recipients (Figure 4A). The reduction in immune cells was attributed to a significant reduction in TAMs following aCSF1 treatment in the presence or absence of aIL34 (Figure 4B). Fluctuation in the numbers of additional myeloid cell populations, such as monocytes/myeloid-derived suppressor cells (MDSCs), was detected as well. Specifically, monocytic MDSCs (mMDSCs; CD11b^+^, Gr-1^int/−^, F4/80^−^) were reduced with aCSF1/IL34 treatment and granulocytic MDSCs (gMDSCs; CD11b^+^, Gr-1^hi^) were increased when aCSF1 was administered (Figure 4C). aIL34 treatment alone did not alter proliferation of TAMs analyzed by cell cycle marker, Ki67 (Figure 4D). CSF1 blockade significantly diminished this parameter in such cells, suggesting that inhibition of myeloid cell proliferation is likely a contributor to curtailed TAM accumulation (Figure 4D). Functional marker analysis within the TAM population revealed significant increases in inflammatory macrophage markers NOS2 and CD86 as well as the anti-inflammatory marker arginase 1 (Arg1) within mice administered with aCSF1 (Figure 4E). In addition, greater than 98.5% of TAMs in all treatment groups expressed MHC class II and the inhibitory ligand, PD-L1 (data not shown); however, the MFI for these markers was significantly elevated in this population when mice received aCSF1 (Figure 4F). Marker expression was not altered by aIL34 administration alone, suggesting that blockade of CSF1, but not IL34, likely altered TAM functionality (Figures 4D,F).

Alterations in TAM content and functionality may influence anti-tumor immune responses; therefore, we evaluated T and NK cell accumulation as well as functional responses. Administration of aCSF1 with or without aIL34 did not significantly influence accumulation of NK cells and CD8^+^ T cells within MC38 tumors (Figure 4G). However, administration of aCSF1, but not aIL34, significantly decreased the number of tumor-resident CD4^+^ T cells (Figure 4G). Characterization of the CD4^+^ compartment revealed that the number of FoxP3^+^ cells, a transcription factor that identifies the regulatory CD4^+^ T cell population, was diminished in mice that received aCSF1, but not aIL34 (Figure 4H). The number of CD4^+^ T cells that were negative for FoxP3, termed T effectors (Teff), was also significantly reduced in mice that were administered both aCSF1 and aIL34, but not aCSF1 alone (Figure 4H). The ratio of the number of CD8^+^ T cells to Tregs was significantly greater in aCSF1-treated mice when compared to control recipients (Figure 4I). Although TAM and Treg content was reduced and TAM functional marker expression was altered in tumors following aCSF1/aIL34 treatment, T cell IFNγ and TNFα production following PMA and ionomycin stimulation and CD8^+^ T cell as well as NK cell intracellular granzyme B content was unchanged (data not shown). Although T cell and NK cell activation was not altered, administration of aCSF1 in combination with aIL34 modestly reduced MC38 tumor growth when compared to control-treated recipients (Figure 4J). In addition to a modest reduction in tumor growth, aCSF1/-IL34 treatment modestly improved survival compared to control IgG recipients (Figure 4J). Anti-PDL1 was superior to aCSF1/aIL34, and the combination of anti-PDL1 and aCSF1/aIL34 was only marginally beneficial (Supplementary Figure 12). In summary, TAM homeostasis within the MC38 tumor microenvironment is maintained by production of CSF1, not IL34, and inhibition of signaling not only influenced the myeloid compartment but also inhibited CD4^+^ T cell accumulation, favoring a reduction in CD4^+^ FoxP3^+^ T cells. Therefore, the modest reduction observed in tumor growth and survival following aCSF1 and aIL34 treatment was likely due to alterations within tumor microenvironment such as inhibition of angiogenesis and/or growth factor production that may be induced by TAM reduction, and not caused by enhanced anti-tumor effector cell activation. Thus, aCSF1 treatment alters mouse tumor microenvironment; however, this treatment alone may not be sufficient to reduce tumor burden.

### Safety of CSF1/IL34 Blockade

#### Prophylactic Treatment of Acsf1/Ail34 Lowers Susceptibility to Listeria Infection Compared to TNF Antagonists

**Figure 5:**
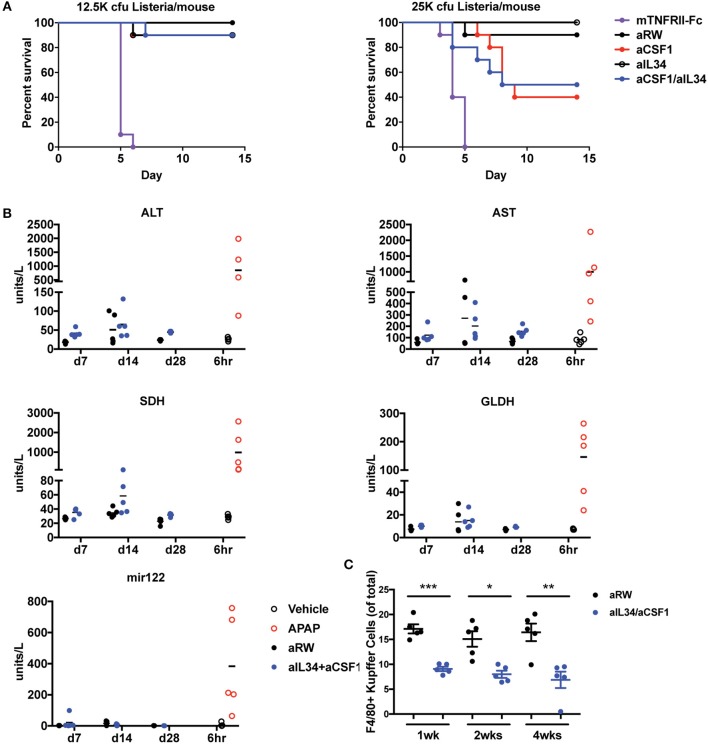
Assessment of infection and liver toxicity risks with aIL34 and/or aCSF1. **(A)** Infection risk: Pre-treatment with aCSF1, aIL34, or aCSF1/aIL34 antibodies prior to *L. monocytogenes* infection resulted in an increase in survival rate compared to TNFRII-Fc. C57BL6 female mice (*n* = 10/group) were treated with aRW, aCSF1, aIL34, aCSF1/aIL34, or mTNFRII-Fc starting 2 days prior to infection. Mice were infected *via* intravenous route with two doses of *L. monocytogenes* [12.5K or 25K colony-forming units (cfu)/mouse] and animals were monitored for 14 days. **(B)** B6C3F1 mice were treated with aRW or a-CSF1/a-IL34 antibodies for 7, 14, or 28 days (*n* = 5/group/time point). Following high-dose APAP (1,200 mg/kg APAP for 6 h), increased ALT, AST, SDH, GLDH, and miR-122 values were observed. Treatment with a-CSF1/a-IL34 antibodies resulted in mild to moderate increases in liver injury biomarkers ALT and AST compared to concurrent anti-RW control values with no significant changes in liver injury biomarkers miR122 or GLDH. Following dosing, animals were necropsied, serum or plasma was collected for liver biomarker analysis, and liver tissue was processed for microscopic evaluation or enumeration of Kupffer cells as described in Materials and Methods. **(C)** Analysis of liver Kupffer cell numbers using F4/80+ IHC. Data in C are displayed as mean ± SEM. ^*^p < 0.05, ^**^p < 0.01, ^***^p < 0.001.

Monocytes and macrophages control bacterial infection and dissemination (46–48). Depletion of macrophage/monocytes for 1 month in adult healthy mice did not reveal any appreciable adverse events and safety issues (Figure 1). Given the central role of macrophages and monocytes in infection, we assessed the consequences of neutralizing CSF1 and/or IL34 in mice infected with *Listeria monocytogen*es. We found that aCSF1/aIL34 blockade and aCSF1-treated mice were susceptible to *Listeria* infection; however, this susceptibility was better tolerated compared to standard of care TNF-FCRII neutralizing agent (Figure 5A). aIL34 did not alter infection susceptibility. We conclude that while CSF1 blockade could be immunosuppressive in the rodent *Listeria* model, there might be a therapeutic window for intervention since other cellular sources of inflammatory cytokines such as T cells or NK cells do not depend on CSF1/IL34 cytokines.

#### Increased Aminotransferases Without Liver Injury as a Result of Decreased Kcs

Therapeutics targeting signaling pathways that promote differentiation, proliferation, and migration of monocytes have been shown to significantly decrease macrophage populations in multiple organs including KCs in the liver. It has been hypothesized that KCs have a role in clearing several serum enzymes, including alanine aminotransferase (ALT) and aspartate aminotransferase (AST); KC reduction is also associated with increased ALT and AST in the absence of hepatocellular injury (49). To evaluate the effects of combination treatment of aCSF1 and aIL34 on KC and liver enzymes, mice were administered aCSF1/aIL34 or an isotype control (aRW), without concomitant liver damage for 7, 14, and 28 days. To compare changes in liver enzymes with a-1CSF1/aIL34 treatment with that of liver injury, a cohort of healthy naïve mice was treated with a high dose of (1200 mg/kg) of acetaminophen (APAP) or vehicle control. Serum or plasma markers of liver injury ALT, AST, sorbitol dehydrogenase (SDH), glutamate dehydrogenase (GLDH), and microRNA-122 (miR-122) were measured at 6 h post-APAP. Additionally, KC numbers and/or liver histopathology was evaluated at several time points in aCSF1/aIL34- or APAP-treated groups, respectively (Figure 5B). Following high-dose APAP treatment, histopathologic examination confirmed the presence of massive zonal to confluent acute hepatocellular necrosis 24 h post-APAP (Supplementary Figure 13). Correlating with liver injury, increased ALT, AST, SDH, GLDH, and miR-122 values were observed in these animals (Figures 5B,C). In mice treated with the CSF1/IL34 blocking antibodies, a 50% decrease in KCs was observed with no histopathologic evidence of liver injury. However, a mild to moderate increase in ALT and AST was observed at all time points evaluated in the aCSF1/IL34 group relative to the concurrent aRW control group. In contrast, miR-122 and GLDH did not increase with decreased KCs with aCSF1/IL34 treatment compared to the aRW control group (Figure 5B). Although no change in SDH was observed at day 7 or 28, high variability was observed at day 14 (Figure 5B). Collectively, these data suggest that aIL34/aCSF1 combination therapy reduces the clearance of liver enzymes ALT and AST as a result of decreased numbers of viable liver KCs. On the other hand, miR-122 and GLDH show no apparent association with KC reduction and, therefore, may be of use as a liver injury biomarker with therapeutics that target macrophages.

## Discussion

CSF1R signaling drives macrophage differentiation to control tissue homeostasis, repair and/or inflammation (5, 50). While analyses of CSF1R, CSF1, or IL34 knockout mice have been informative, developmental defects limit the utility of such models at both steady state and under inflammatory conditions (20–25). In this study, we used IL34 and CSF1 blocking antibodies to study various tissue macrophages in the skin (LCs), gut, lung, liver (KCs), brain (microglia), kidney, and lymphoid organs. Except for lung alveolar macrophages, CSF1 plays a dominant role in regulating most tissue macrophages except for microglia, LCs, and kidney macrophages, which differentially depend on IL34 or both IL34/CSF1. Our findings with the blockade of CSF1, IL34, or both are consistent with studies in CSF1^OP/OP^ mice, IL34-knockout (23), and CSF1R receptor knockout mice (23, 51), suggesting that during development, CSF1 and IL34 play non-redundant roles in macrophage differentiation and maintenance in different tissues.

Current data suggest that restricted expression of IL34 and CSF1 localize the function of CSF1R to promote the differentiation of certain macrophages including microglia, LC, or kidney macrophages. Analysis of several preclinical inflammatory disease models shows that IL34 and CSF1 expression might be dysregulated in inflammatory diseases. We report here that depending on the disease, single or dual blockade of IL34 and CSF1 is needed for controlling inflammation consistent with the concordant tissue-specific expression of both cytokines in diseases such as colitis or arthritis. In murine arthritis models, CSF1 appears to be the major driver of efficacy and macrophage differentiation in CIA or TNFΔ^ARE^. However, in DSS colitis or TNFΔ^ARE^ ileitis, the blockade of CSF1 and IL34 is most beneficial. Our studies suggest that targeting only IL34 or CSF1 may not be adequate to control inflammation in certain disease conditions. A bispecific platform targeting both IL34 and CSF1 should be considered for IBD. Consistent with these results, soluble CSF1 receptor has been reported to inhibit macrophage proliferation (52, 53). In our studies, both CSF1 and TNF antagonist strategies confer comparable efficacy. Additional studies are needed to determine whether combination of the two drug modalities provides better efficacy in CIA or colitis models.

Blockade of CSF1/IL34 could increase the risk of infections due to a broad decrease in macrophage populations in multiple organs; however, we did not observe worse outcomes in a *Listeria* infection model compared to anti-TNF therapy, suggesting that risk of infection may also be reduced in clinical settings. Due to aIL34/aCSF1 effects on KC, reduced clearance of liver enzymes has been observed in cynomolgus monkeys and humans, as well as in our mouse studies. An increase in liver enzyme activity not due to hepatocellular injury compromises clinical monitoring for liver injury. Our initial studies assessing additional markers of liver injury suggest that miR-122 or GLDH may be of use as a liver biomarker to monitor liver injury with aIL34/aCSF1 or other therapeutics targeting macrophages (54, 55). However, use of these markers for this purpose in clinical setting would require further validation in both preclinical and clinical setting.

We utilized the MC38 tumor model to study how systemic inhibition of CSF1 and/or IL34 impacts the tumor microenvironment and growth. Our analysis suggests that CSF1 primarily drives myeloid cell content within the MC38 tumor microenvironment. The MC38 tumor model is partially responsive to anti-PDL1 and other immunotherapies and is heavily infiltrated with myeloid cells (45). Previous studies have demonstrated that TAM homeostasis requires CSF1R signaling where antibody blockade of CSF1R dramatically decreased TAM tumor content (56). Given that anti-CSF1R blocks both CSF1 and IL34 binding and MC38 tumor cells express both these cytokines, our studies reveal that TAM homeostasis is primarily dependent on CSF1. The dramatic reduction in TAM tumor content was likely mediated by reduced monocyte recruitment as well as diminished macrophage differentiation, survival, and proliferation as demonstrated by reduced Ki67 staining. Although TAM numbers were significantly reduced with CSF1 blockade, they exhibited a more activated phenotype that may be due to increased inflammation within the tumor as was shown with a CSF1R inhibitor (57). Consistent with this, a significant reduction in regulatory T cells was observed, but this did not translate into increased numbers of CD8^+^ T or NK cells. Presumably, combining anti-CSF1 treatment with checkpoint blockade such as *via* anti-PD1 and anti-CTLA4 antibodies may enhance immune cell anti-tumor activity and improved efficacy over single-agent treatment as was demonstrated in established pancreatic ductal adenocarcinoma tumors (57). Translational proof of concept may be provided shortly since clinical trials are underway exploring CSF1R-targeted agents in combination with checkpoint inhibitors (58). Nevertheless, additional studies are needed to understand macrophage heterogeneity and function within the tumor microenvironment as well as how selective blockade of CSF1, IL34, or CSF1R and subsequent effects on TAMs correspond with response to checkpoint blockade.

Therapeutic targeting of CSF1R axes is under intense investigation focusing on either development of small molecules designed to block the tyrosine kinase activity of CSF1R or human monoclonal antibodies designed to target CSF1R to prevent the binding of both CSF1 and IL34 ligands (58). CSF1R kinase inhibitors, such as PLX-3397 (pexidartinib), ABT-869 (linifanib), ABT-869, and BLZ-94, are at various stages in clinical investigation (59). Anti-CSF1R blocking antibodies, such as cabiralizumab (humanized IgG4), RG-7155 (humanized IgG1), IMC-CS4 (human IgG1), and AMG-820 (human IgG2), are also being evaluated in clinical trials as single agents or in combination with other therapeutics (59). While encouraging in PVNS patients (60, 61), the outcomes with CSF1R inhibitors as a monotherapy in solid tumors have been disappointing. Current efforts are aimed at combining CSF1R inhibitors with immunotherapy to potentiate and enhance tumor immunity (59).

Our studies reveal the utility and complexity of single or combination therapeutics against CSF1 or IL34 as a bi-specific drug in inflammation or cancer. Targeting CSF1 and/or IL34 ligands vs. CSF1R may be beneficial depending on the indication to avoid systemic inhibition and reduce adverse effects. In microglia-centric neuro-inflammatory models, IL34 may be examined as a drug candidate; however, in certain tumors, single blockade of CSF1 might be desirable. In addition, targeting ligands might provide better drug penetration and subsequent antagonist activity. Our data also reveal novel insights into distinct subsets of tissue macrophages (e.g., kidney) that may uniquely require CSF1 and/or IL34 for their differentiation. On-going studies using high-resolution gene expression analyses of macrophage/monocyte subsets found in inflammatory or tumor tissues will better inform future novel therapeutics.

## Data Availability

All datasets generated for this study are included in the manuscript and the Supplementary Files.

## Author Contributions

WLi, KS, YS, and JL executed animal mechanistic studies. CA, LD, PC, and NR performed IHC pathology and histology studies. MX, JL, MB, JD, ZH, JZ, SJ, and ES executed animal studies. WLi, SR, CL, JY, DDe, JE, EM, and HN performed different assays. KBar, VG, RC, and RW performed imaging. YC, W-CL, and YW generated antibodies. JM, KW, and PA did tumor studies. DM, TL, DDa, PK, ED, HU, and KA planed and did safety studies. MK performed IBD gene expression studies. AZ, DX, WLi, KA, JY, KW, KBao, CA, PC, AH, and CC wrote the manuscript with major contributions from AZ, WLi, and DX. AZ and FM conceived the idea and supervised the studies. All authors read, edited, and approved the manuscript.

### Conflict of Interest Statement

All studies were funded by Genentech. All authors were employees of Genentech, Inc., a member of the Roche group at the time when studies were done. The funders had no role in study design, data collection and analysis, decision to publish, or preparation of the manuscript. The authors declare no other competing financial interests. AZ is currently an employee of TRex Bio. FM and JD are currently employees of Amgen. LD is an employee of Gilead Sciences. PA is an employee of Adaptive Biotechnologies. DM is currently working at Aligos Therapeutics. KA is employee of Ultragenyx Pharmaceutical. DDa is employee of Danilenko Nonclinical Drug Development, LLC.

## Supplementary Material

Supplementary Figure 1 CSF1 and IL34 neutralizing antibodies. **(A)** Impact of aCSF1 or aIL34 on blocking the proliferation of murine MNFS-60 cell line *via* IL34 (50 ng/ml) or CSF1 (50 ng/ml) cytokines. IL34 50% effective concentration (EC_50_) was determined as 24.8 ng/ml whereas CSF1 EC_50_ was 0.6 ng/ml. The 50% maximum inhibitory concentration (IC_50_) of aIL34 was determined as 30 ng/ml whereas aCSF1 was 1.1 μg/ml. **(B)** PK properties of aCSF1 or aIL34 antibodies. Concentration of aCSF1 or aIL34 antibodies in plasma was determined as described in methods.

Supplementary Figure 2 Impact of CSF1 and or IL34 blockade in circulating monocytes. Steady-state CD11b^+^CX_3_CR1^hi^, CD11b^+^CX_3_CR1^int^, and CD11b^+^CX_3_CR1^neg^ populations in **(A)** blood, **(B)** spleen, **(C)** kidney, and **(D)** colon with aIL34 or aCSF11 blockade or combination blockade in control with aRW. Data are displayed as Mean ± SEM. ^*^*p* < 0.05, ^***^*p* < 0.001, ^****^*p* < 0.0001.

Supplementary Figure 3 CSF1 and IL34 expression in inflammatory diseases. **(A)** CSF1 and IL34 are elevated in human arthritis. The serum from healthy donors (Control, CTL, *n* = 42), rheumatoid arthritis (RA) patients (*n* = 225), osteoarthritis (OA) patients (*n* = 23), cell-free synovial fluid (SF) from RA (*n* = 14), and SF from OA (*n* = 17) were assessed by ELISA. **(B)** Circulating mouse IL34 but not CSF1 is increased in mouse CIA (*n* = 10). **(C)** Analysis of IL34, CSF1, CSF1R, CCL2, and CCR2 in IBD compared to non-IBD diverticulitis (DVT) or normal tissues adjacent to tumor controls (NAT). **(D)** Elevated CSF1 and IL34 in DSS colitis gut epithelial injury model. **(E)** Increased CSF1 or IL34 in different parts of intestinal tissues in TNFΔ^ARE^. Different tissues were cultured overnight and supernatants were assayed by ELISA. CSF1 is elevated in ileum and cecum whereas IL34 is elevated in various areas of the gut including duodenum, jejunum, ileum, colon, and cecum. ^*^*p* < 0.05, ^***^*p* < 0.001, ^****^*p* < 0.0001.

Supplemental Figure 4 Cartilage injury. **(A)** Cartilage injury score of CIA model. **(B)** Cartilage histology and microCT correlation. Animals are treated with aRW, TNFRII-Fc, or aIL34, and aCSF11 combination blockade antibodies. Data are displayed as mean ± SEM. ^*^*p* < 0.05.

Supplementary Figure 5 Increased macrophages in hind paws in TNFΔ^ARE^ disease model. IHC staining tissue macrophage with rat anti-mouse F4/80 showed significantly increased macrophage number in TNFΔ^ARE^ mice in hind paws (19 weeks of age). ^***^*p* < 0.001.

Supplementary Figure 6 FACS analysis and numbers of colon macrophages with aCSF1 and/or IL34 blockade using CX3CR1^wt/gfp^ reporter model in DSS model. DSS-treated animals were treated with aRW, aIL34, or CSF1 alone or with combination blockade antibodies. Data are displayed as mean ± SEM. ^*^*p* < 0.05, ^**^*p* < 0.01.

Supplementary Figure 7 Consequence of CSF1 and/or IL34 blockade on blood, mLN or splenic macrophages in DSS colitis model. Naïve C57BL6 or DSS colitis mice treated with aRW, aIL34, aCSF1, or aIL34/aCSF1. **(A)** Circulating monocytes in blood. **(B)** Macrophages in mesenteric lymph nodes and spleen. Data are displayed as Mean ± SEM. ^**^*p* < 0.01, ^***^*p* < 0.001, ^****^*p* < 0.0001.

Supplementary Figure 8 Increased macrophages in ileum in TNFΔ^ARE^ disease model. IHC staining tissue macrophage with rat anti-mouse F4/80 showed significantly increased macrophage number in TNFΔ^ARE^ mice in ileum (19 weeks of age). ^**^*p* < 0.01.

Supplementary Figure 9 Impact of IL34 or/CSF1 blockade on accelerated NZB/W F1 lupus. **(A)** IFNα-accelerated model: adenovirus-5 (Ad5)-IFNα or Ad5-LacZ control viral vectors were administered by intravenous injection into 12-week-old NZB/WF1 female mice. Three weeks post Ad5 viral vector injection aCSF1 or aIL34 or in combination was dosed at 10 mg/kg subcutaneously twice per week for 8 weeks. Cyclophosphamide (CYC; Baxter) was used as a reference treatment. **(B)** Pristane-accelerated: Pristane was administered to 3-months-old female NZB/W F1 mice, and after 2 months, treatments were started with aCSF1/IL34, aRW or Cytoxan.

Supplementary Figure 10 CSF1, IL34, and CSF1R message expression in various cancers.

Supplementary Figure 11 *In vitro* secretion of CSF1 and IL34 by murine tumor lines.

Supplementary Figure 12 Impact of a-PDL1 and aCSF1/aI-L34 combination treatment on MC38 tumor volume. **(A)** Average tumor volume over time; **(B)** individual mouse tumor growth curves.

Supplementary Figure 13 Massive zonal to confluent acute hepatocellular necrosis 24 h post-APAP. H & E stain.
