# Supplementary figures and images for: Function of CSF1 and IL34 in Macrophage Homeostasis, Inflammation, and Cancer
Source: Front Immunol. 2019 Sep 4;10:2019. doi: 10.3389/fimmu.2019.02019 (PMC6736990; doi:10.3389/fimmu.2019.02019)

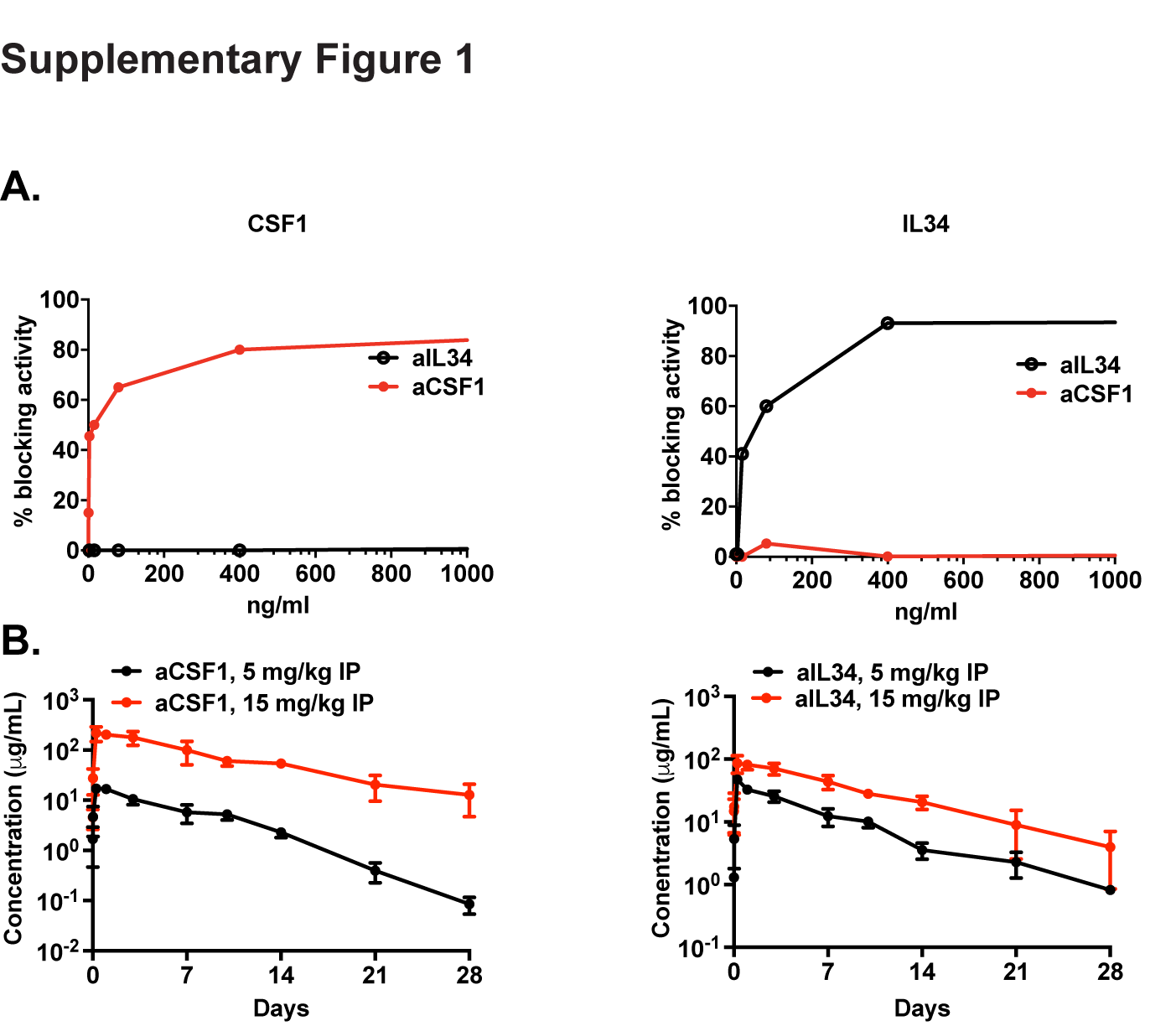

Supplement: Supplementary Figure 1 — CSF1 and IL34 neutralizing antibodies. (A) Impact of aCSF1 or aIL34 on blocking the proliferation of murine MNFS-60 cell line via IL34 (50 ng/ml) or CSF1 (50 ng/ml) cytokines. IL34 50% effective concentration (EC50) was determined as 24.8 ng/ml whereas CSF1 EC50 was 0.6 ng/ml. The 50% maximum inhibitory concentration (IC50) of aIL34 was determined as 30 ng/ml whereas aCSF1 was 1.1 μg/ml. (B) PK properties of aCSF1 or aIL34 antibodies. Concentration of aCSF1 or aIL34 antibodies in plasma was determined as described in methods. [file Data_Sheet_1.zip › Supp Figure1.tif]

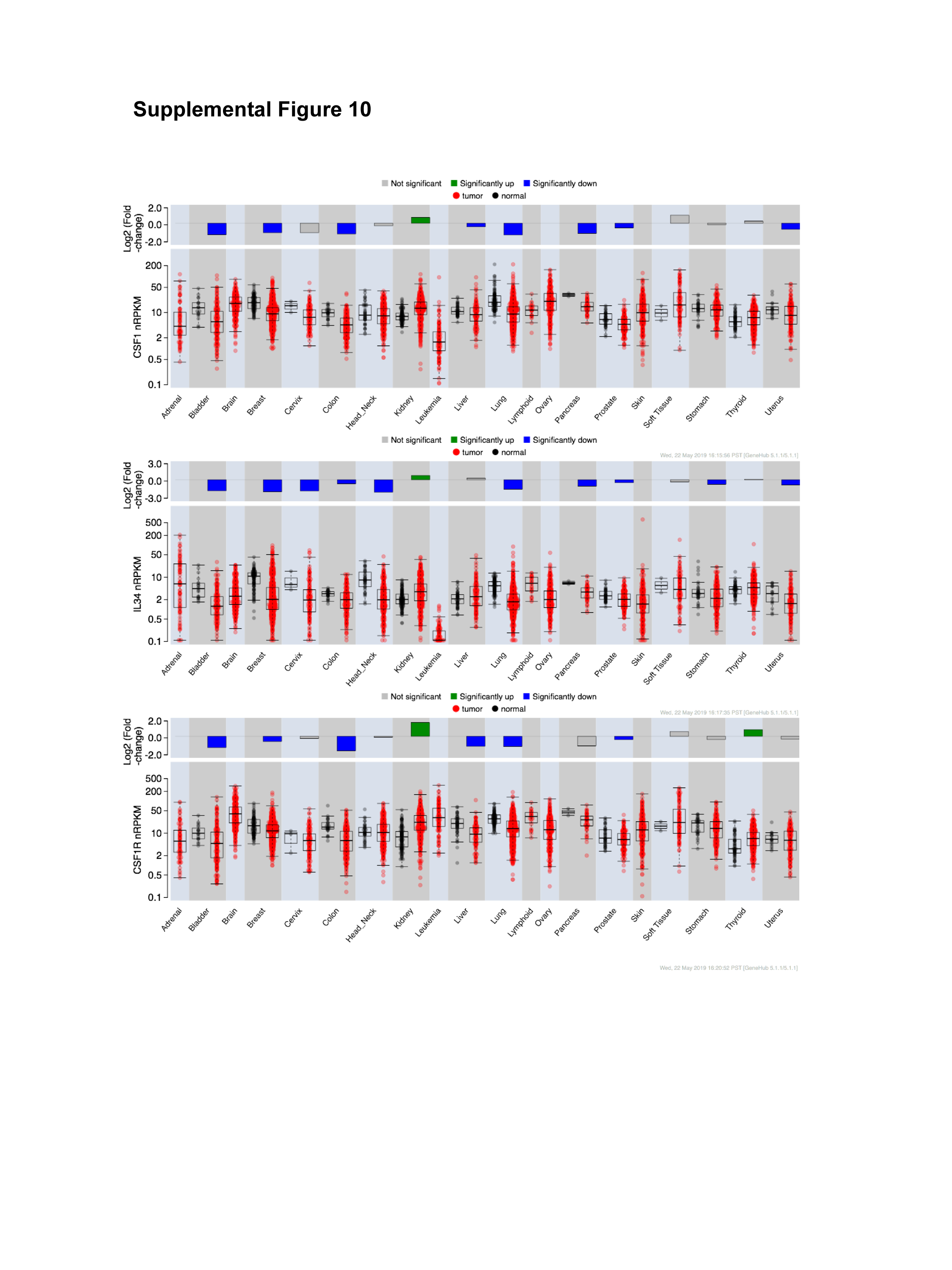

Supplement: Supplementary Figure 1 — CSF1 and IL34 neutralizing antibodies. (A) Impact of aCSF1 or aIL34 on blocking the proliferation of murine MNFS-60 cell line via IL34 (50 ng/ml) or CSF1 (50 ng/ml) cytokines. IL34 50% effective concentration (EC50) was determined as 24.8 ng/ml whereas CSF1 EC50 was 0.6 ng/ml. The 50% maximum inhibitory concentration (IC50) of aIL34 was determined as 30 ng/ml whereas aCSF1 was 1.1 μg/ml. (B) PK properties of aCSF1 or aIL34 antibodies. Concentration of aCSF1 or aIL34 antibodies in plasma was determined as described in methods. [file Data_Sheet_1.zip › Supp Figure10.tif]

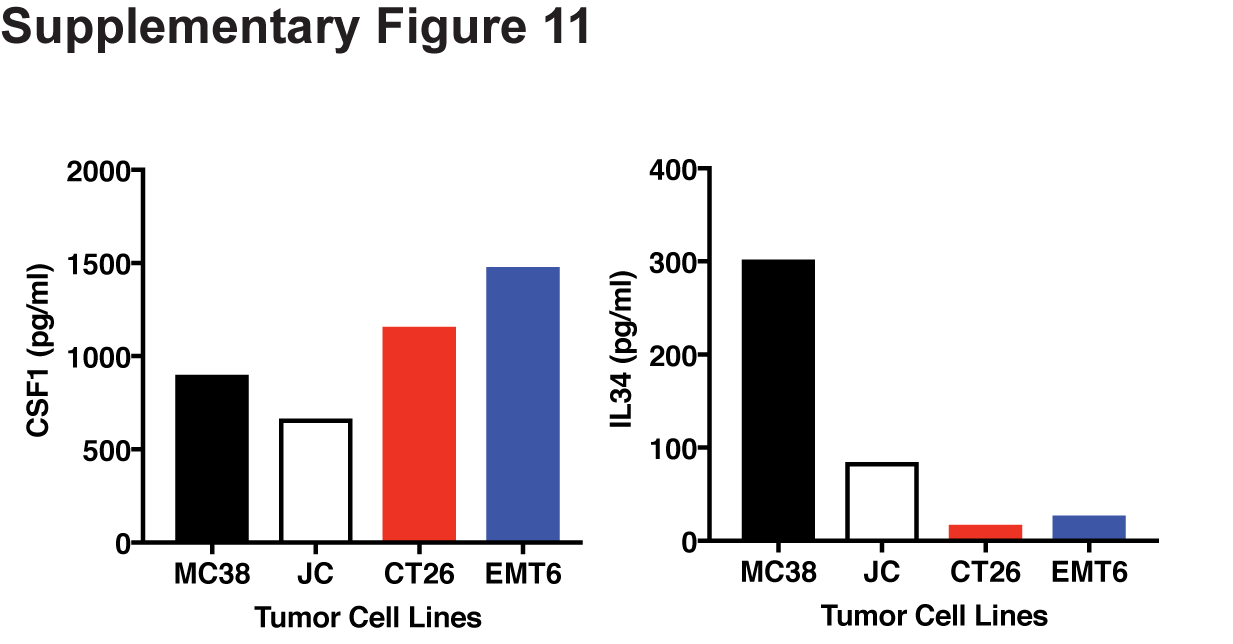

Supplement: Supplementary Figure 1 — CSF1 and IL34 neutralizing antibodies. (A) Impact of aCSF1 or aIL34 on blocking the proliferation of murine MNFS-60 cell line via IL34 (50 ng/ml) or CSF1 (50 ng/ml) cytokines. IL34 50% effective concentration (EC50) was determined as 24.8 ng/ml whereas CSF1 EC50 was 0.6 ng/ml. The 50% maximum inhibitory concentration (IC50) of aIL34 was determined as 30 ng/ml whereas aCSF1 was 1.1 μg/ml. (B) PK properties of aCSF1 or aIL34 antibodies. Concentration of aCSF1 or aIL34 antibodies in plasma was determined as described in methods. [file Data_Sheet_1.zip › Supp Figure11 .tif]

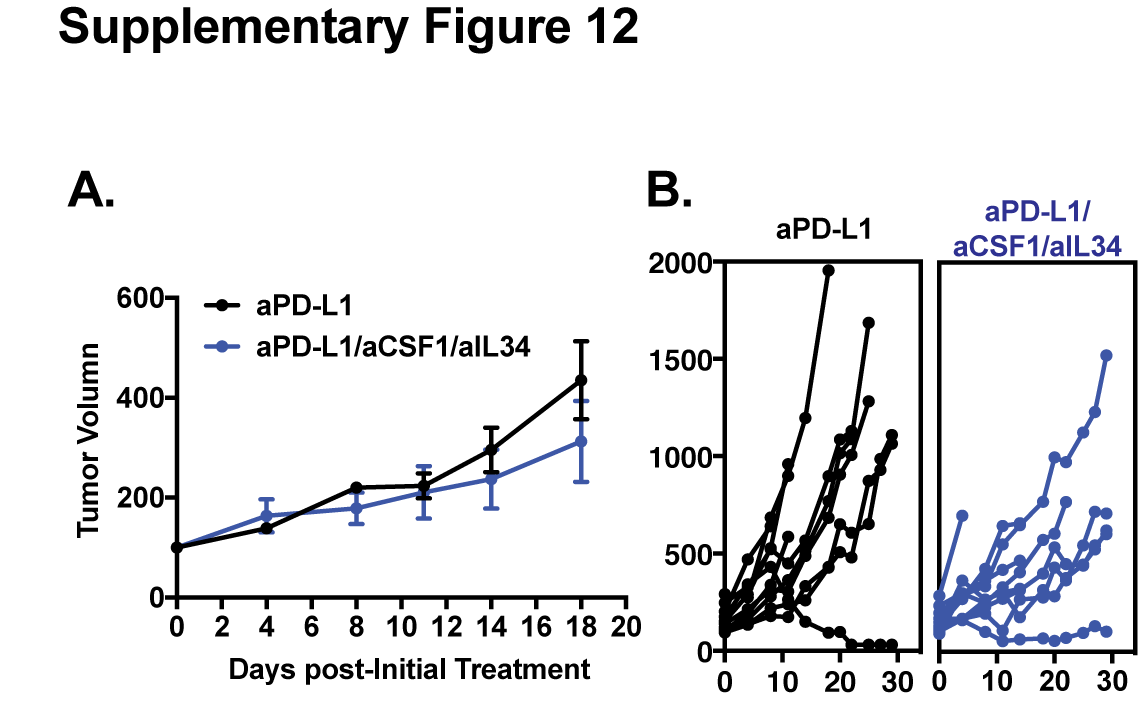

Supplement: Supplementary Figure 1 — CSF1 and IL34 neutralizing antibodies. (A) Impact of aCSF1 or aIL34 on blocking the proliferation of murine MNFS-60 cell line via IL34 (50 ng/ml) or CSF1 (50 ng/ml) cytokines. IL34 50% effective concentration (EC50) was determined as 24.8 ng/ml whereas CSF1 EC50 was 0.6 ng/ml. The 50% maximum inhibitory concentration (IC50) of aIL34 was determined as 30 ng/ml whereas aCSF1 was 1.1 μg/ml. (B) PK properties of aCSF1 or aIL34 antibodies. Concentration of aCSF1 or aIL34 antibodies in plasma was determined as described in methods. [file Data_Sheet_1.zip › Supp Figure12.tif]

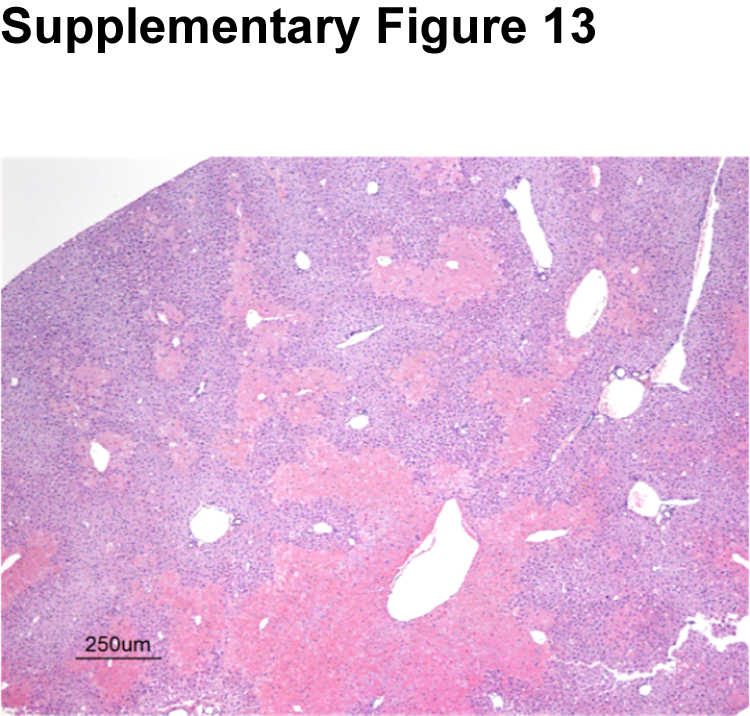

Supplement: Supplementary Figure 1 — CSF1 and IL34 neutralizing antibodies. (A) Impact of aCSF1 or aIL34 on blocking the proliferation of murine MNFS-60 cell line via IL34 (50 ng/ml) or CSF1 (50 ng/ml) cytokines. IL34 50% effective concentration (EC50) was determined as 24.8 ng/ml whereas CSF1 EC50 was 0.6 ng/ml. The 50% maximum inhibitory concentration (IC50) of aIL34 was determined as 30 ng/ml whereas aCSF1 was 1.1 μg/ml. (B) PK properties of aCSF1 or aIL34 antibodies. Concentration of aCSF1 or aIL34 antibodies in plasma was determined as described in methods. [file Data_Sheet_1.zip › Supp Figure13.tif]

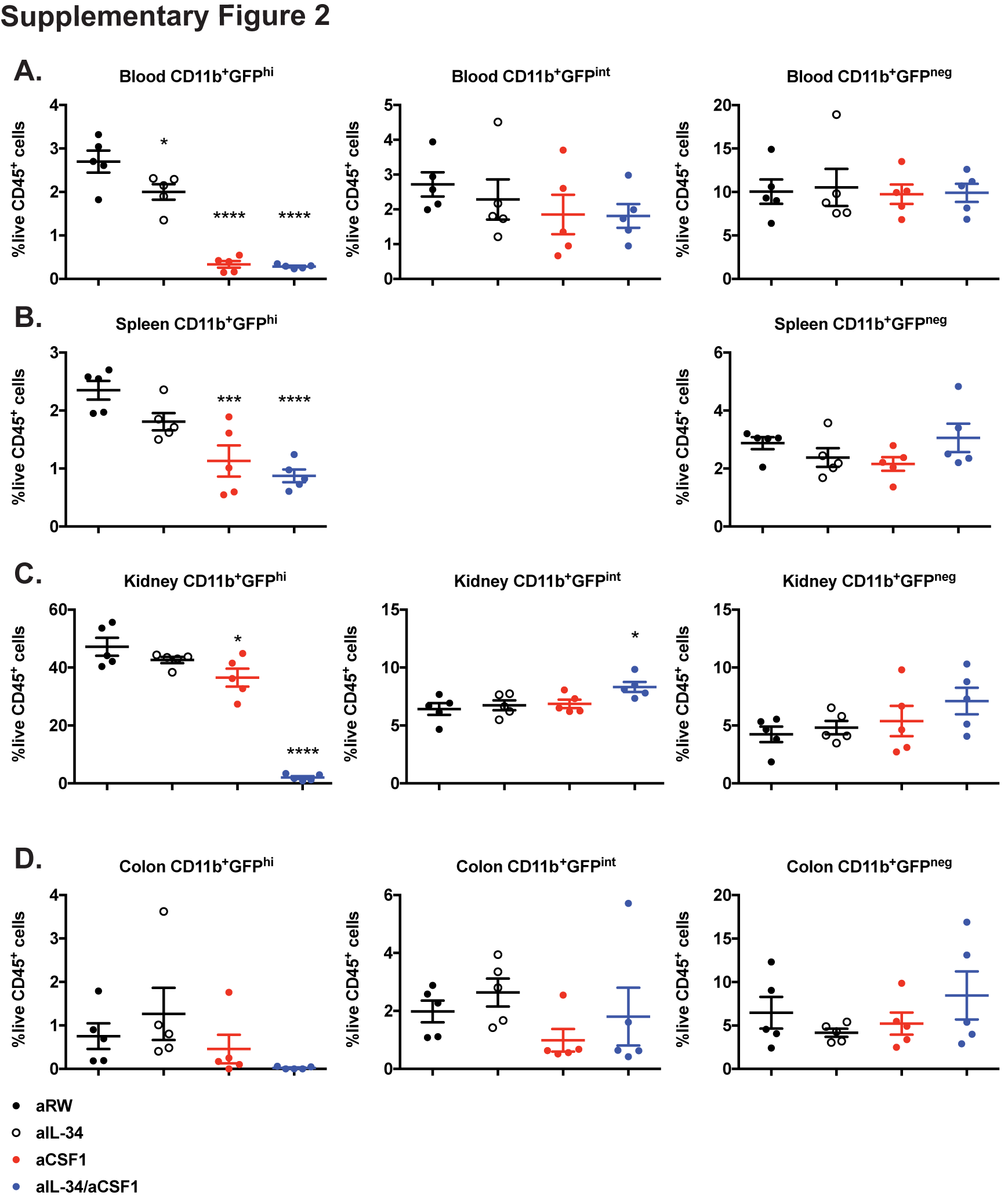

Supplement: Supplementary Figure 1 — CSF1 and IL34 neutralizing antibodies. (A) Impact of aCSF1 or aIL34 on blocking the proliferation of murine MNFS-60 cell line via IL34 (50 ng/ml) or CSF1 (50 ng/ml) cytokines. IL34 50% effective concentration (EC50) was determined as 24.8 ng/ml whereas CSF1 EC50 was 0.6 ng/ml. The 50% maximum inhibitory concentration (IC50) of aIL34 was determined as 30 ng/ml whereas aCSF1 was 1.1 μg/ml. (B) PK properties of aCSF1 or aIL34 antibodies. Concentration of aCSF1 or aIL34 antibodies in plasma was determined as described in methods. [file Data_Sheet_1.zip › Supp Figure2.tif]

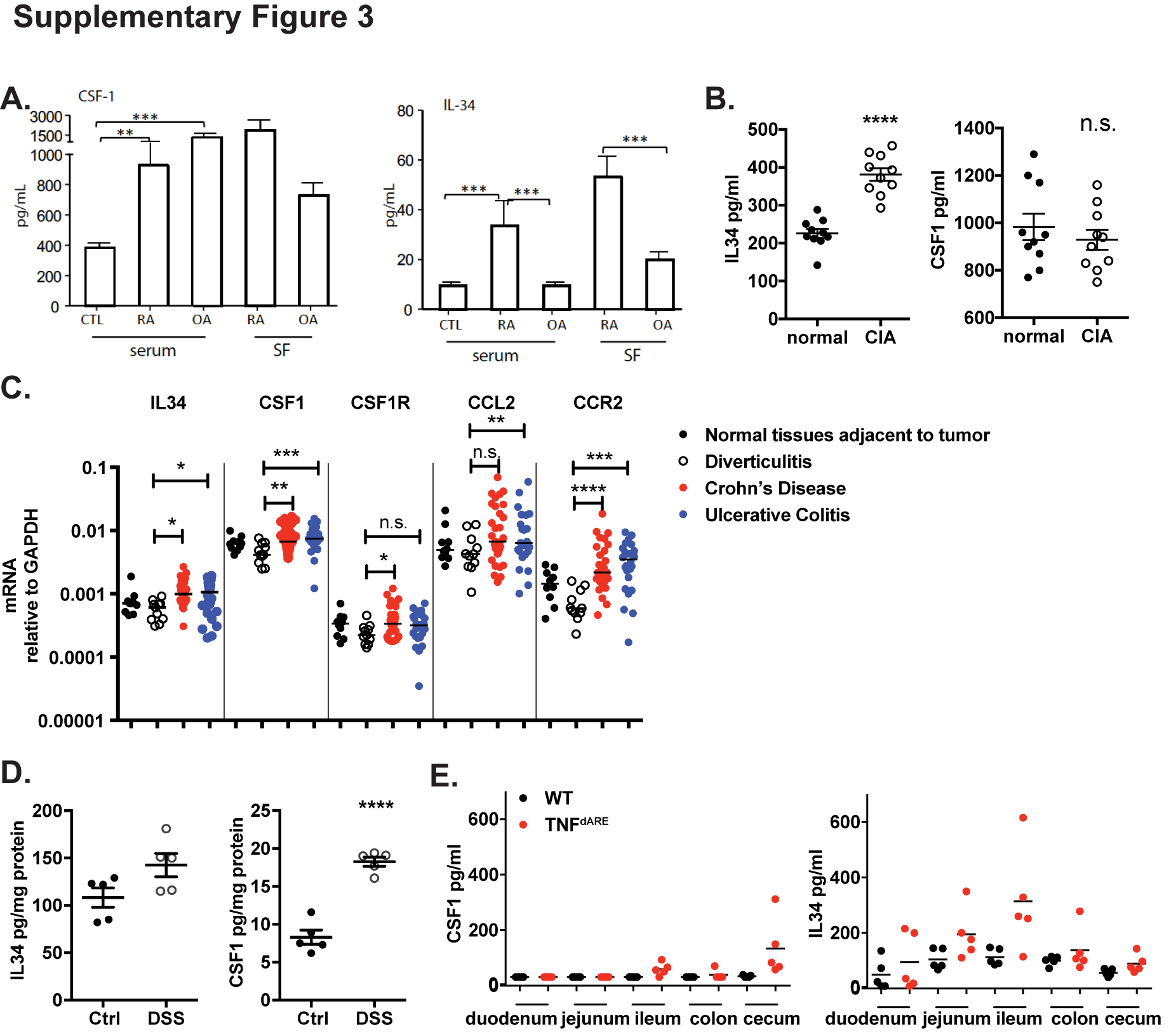

Supplement: Supplementary Figure 1 — CSF1 and IL34 neutralizing antibodies. (A) Impact of aCSF1 or aIL34 on blocking the proliferation of murine MNFS-60 cell line via IL34 (50 ng/ml) or CSF1 (50 ng/ml) cytokines. IL34 50% effective concentration (EC50) was determined as 24.8 ng/ml whereas CSF1 EC50 was 0.6 ng/ml. The 50% maximum inhibitory concentration (IC50) of aIL34 was determined as 30 ng/ml whereas aCSF1 was 1.1 μg/ml. (B) PK properties of aCSF1 or aIL34 antibodies. Concentration of aCSF1 or aIL34 antibodies in plasma was determined as described in methods. [file Data_Sheet_1.zip › Supp Figure3.tif]

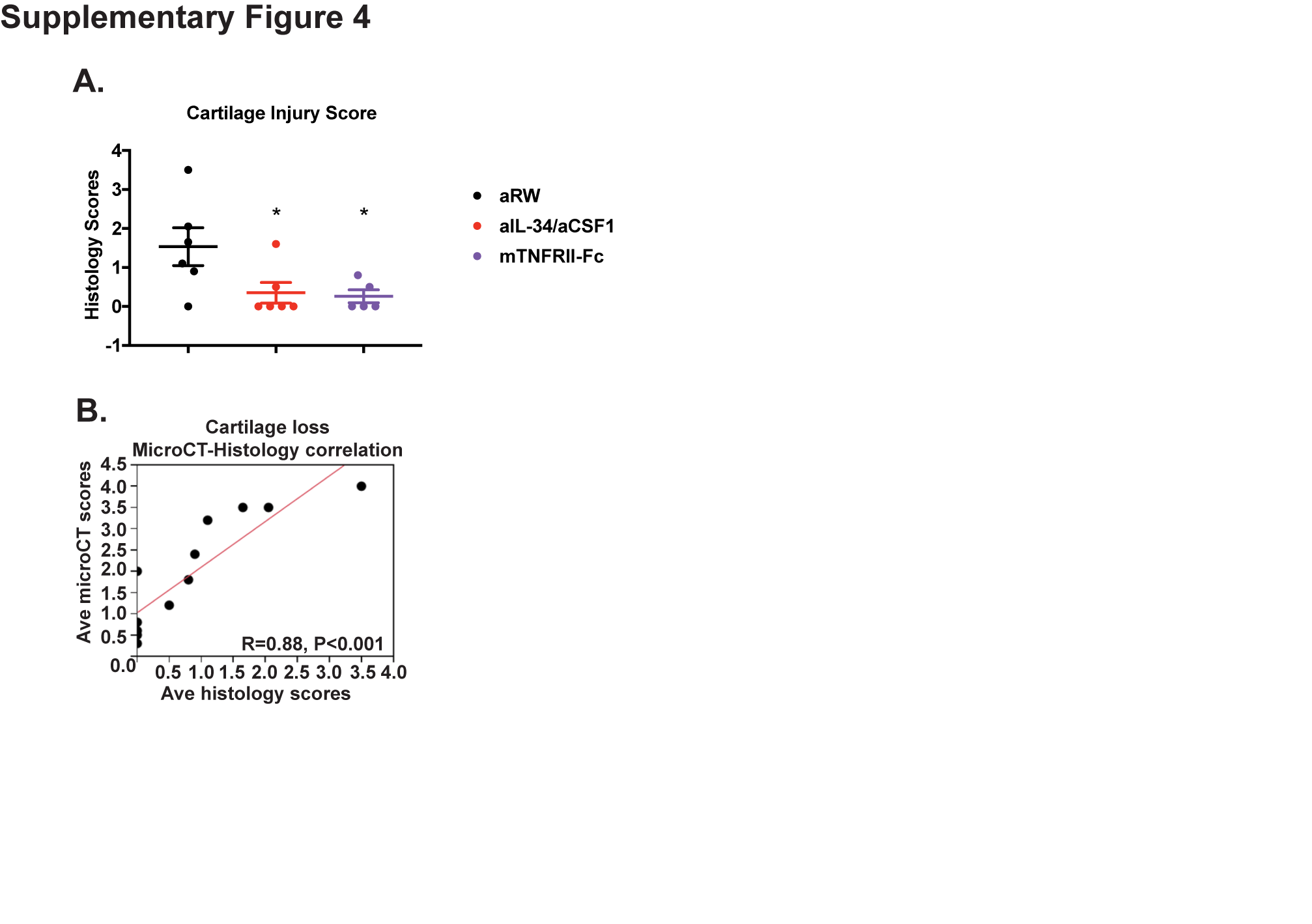

Supplement: Supplementary Figure 1 — CSF1 and IL34 neutralizing antibodies. (A) Impact of aCSF1 or aIL34 on blocking the proliferation of murine MNFS-60 cell line via IL34 (50 ng/ml) or CSF1 (50 ng/ml) cytokines. IL34 50% effective concentration (EC50) was determined as 24.8 ng/ml whereas CSF1 EC50 was 0.6 ng/ml. The 50% maximum inhibitory concentration (IC50) of aIL34 was determined as 30 ng/ml whereas aCSF1 was 1.1 μg/ml. (B) PK properties of aCSF1 or aIL34 antibodies. Concentration of aCSF1 or aIL34 antibodies in plasma was determined as described in methods. [file Data_Sheet_1.zip › Supp Figure4.tif]

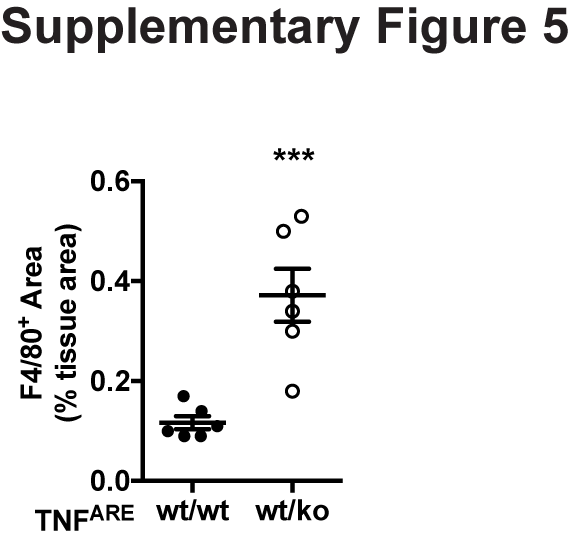

Supplement: Supplementary Figure 1 — CSF1 and IL34 neutralizing antibodies. (A) Impact of aCSF1 or aIL34 on blocking the proliferation of murine MNFS-60 cell line via IL34 (50 ng/ml) or CSF1 (50 ng/ml) cytokines. IL34 50% effective concentration (EC50) was determined as 24.8 ng/ml whereas CSF1 EC50 was 0.6 ng/ml. The 50% maximum inhibitory concentration (IC50) of aIL34 was determined as 30 ng/ml whereas aCSF1 was 1.1 μg/ml. (B) PK properties of aCSF1 or aIL34 antibodies. Concentration of aCSF1 or aIL34 antibodies in plasma was determined as described in methods. [file Data_Sheet_1.zip › Supp Figure5.tif]

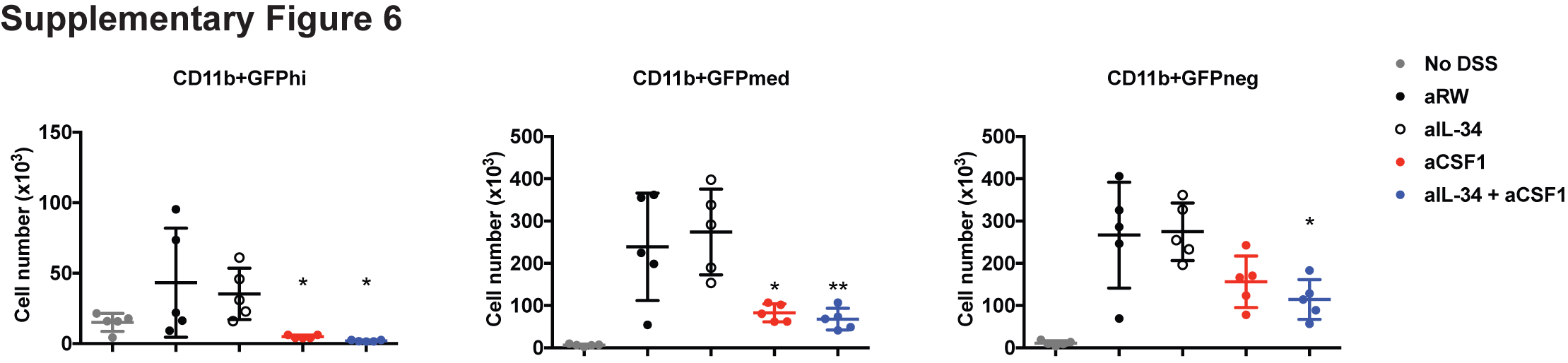

Supplement: Supplementary Figure 1 — CSF1 and IL34 neutralizing antibodies. (A) Impact of aCSF1 or aIL34 on blocking the proliferation of murine MNFS-60 cell line via IL34 (50 ng/ml) or CSF1 (50 ng/ml) cytokines. IL34 50% effective concentration (EC50) was determined as 24.8 ng/ml whereas CSF1 EC50 was 0.6 ng/ml. The 50% maximum inhibitory concentration (IC50) of aIL34 was determined as 30 ng/ml whereas aCSF1 was 1.1 μg/ml. (B) PK properties of aCSF1 or aIL34 antibodies. Concentration of aCSF1 or aIL34 antibodies in plasma was determined as described in methods. [file Data_Sheet_1.zip › Supp Figure6.tif]

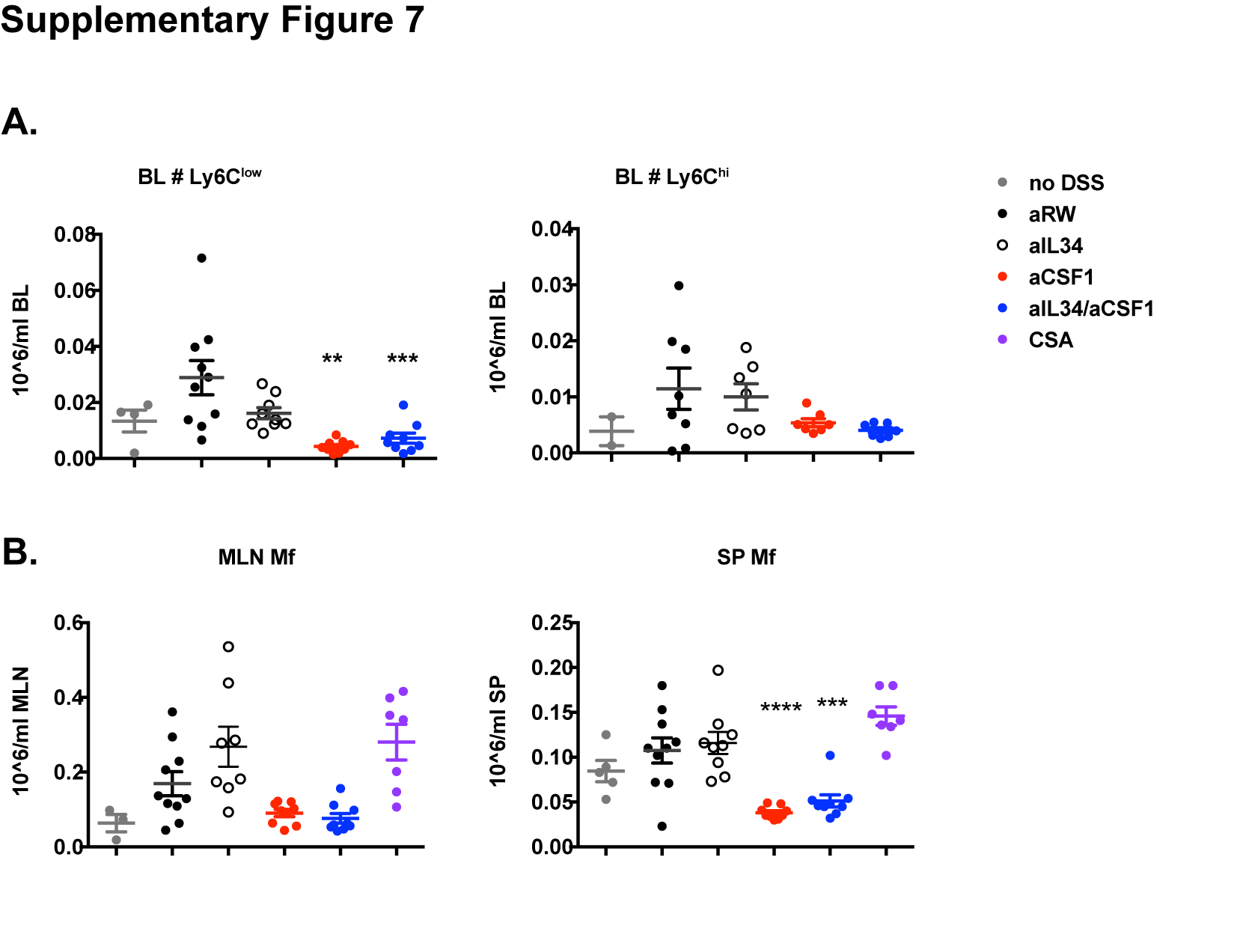

Supplement: Supplementary Figure 1 — CSF1 and IL34 neutralizing antibodies. (A) Impact of aCSF1 or aIL34 on blocking the proliferation of murine MNFS-60 cell line via IL34 (50 ng/ml) or CSF1 (50 ng/ml) cytokines. IL34 50% effective concentration (EC50) was determined as 24.8 ng/ml whereas CSF1 EC50 was 0.6 ng/ml. The 50% maximum inhibitory concentration (IC50) of aIL34 was determined as 30 ng/ml whereas aCSF1 was 1.1 μg/ml. (B) PK properties of aCSF1 or aIL34 antibodies. Concentration of aCSF1 or aIL34 antibodies in plasma was determined as described in methods. [file Data_Sheet_1.zip › Supp Figure7.tif]

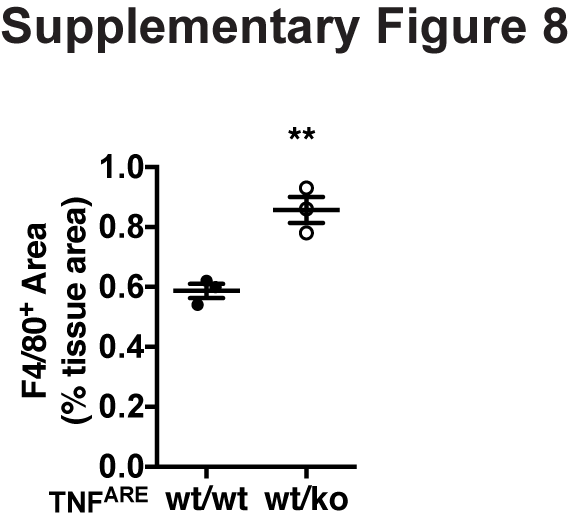

Supplement: Supplementary Figure 1 — CSF1 and IL34 neutralizing antibodies. (A) Impact of aCSF1 or aIL34 on blocking the proliferation of murine MNFS-60 cell line via IL34 (50 ng/ml) or CSF1 (50 ng/ml) cytokines. IL34 50% effective concentration (EC50) was determined as 24.8 ng/ml whereas CSF1 EC50 was 0.6 ng/ml. The 50% maximum inhibitory concentration (IC50) of aIL34 was determined as 30 ng/ml whereas aCSF1 was 1.1 μg/ml. (B) PK properties of aCSF1 or aIL34 antibodies. Concentration of aCSF1 or aIL34 antibodies in plasma was determined as described in methods. [file Data_Sheet_1.zip › Supp Figure8.tif]

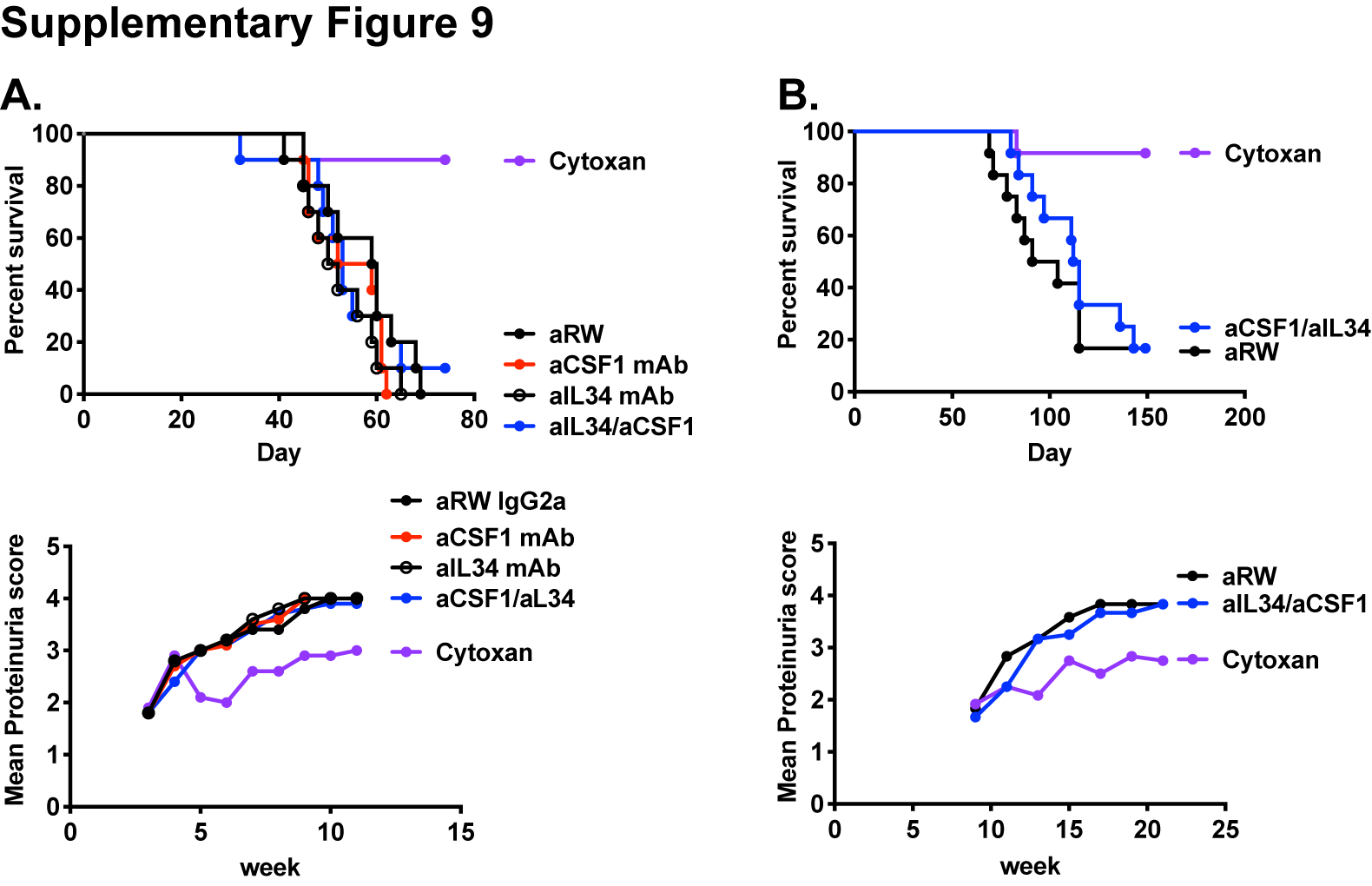

Supplement: Supplementary Figure 1 — CSF1 and IL34 neutralizing antibodies. (A) Impact of aCSF1 or aIL34 on blocking the proliferation of murine MNFS-60 cell line via IL34 (50 ng/ml) or CSF1 (50 ng/ml) cytokines. IL34 50% effective concentration (EC50) was determined as 24.8 ng/ml whereas CSF1 EC50 was 0.6 ng/ml. The 50% maximum inhibitory concentration (IC50) of aIL34 was determined as 30 ng/ml whereas aCSF1 was 1.1 μg/ml. (B) PK properties of aCSF1 or aIL34 antibodies. Concentration of aCSF1 or aIL34 antibodies in plasma was determined as described in methods. [file Data_Sheet_1.zip › Supp Figure9.tif]
